# SHPRH together with the Ube2D family of enzymes directly ubiquitinates PCNA at Lys164 *in vitro*

**DOI:** 10.1371/journal.pone.0347227

**Published:** 2026-04-16

**Authors:** Xinyang Wang, Yixiong Hu, Yajiao Wen, Miaomiao Shen, Pu Chen, Song Xiang

**Affiliations:** 1 Department of Biochemistry and Molecular Biology, Key Laboratory of Immune Microenvironment and Disease (Ministry of Education), The province and ministry co-sponsored collaborative innovation center for medical epigenetics, State Key Laboratory of Experimental Hematology, Tianjin Medical University, Tianjin, P. R. CHINA; 2 Tianjin Medical University Cancer Institute & Hospital, National Clinical Research Center for Cancer, Tianjin’s Clinical Research Center for Cancer, Key Laboratory of Cancer Prevention and Therapy, Tianjin, P. R. CHINA; University of Nova Gorica, SLOVENIA

## Abstract

Ubiquitin-chain attachment to a highly conserved Lys164 residue in the proliferating cell nuclear antigen (PCNA) signals for template switching events to resume stalled DNA replication. In human cells, the reaction is catalyzed by the ubiquitin ligases SNF2 histone linker PHD RING helicase (SHPRH) or helicase like transcription factor (HLTF), together with the Ubc13-Mms2 ubiquitin conjugating enzyme complex. The reaction requires mono-ubiquitinated PCNA as the substrate. Here, we provide biochemical evidence that SHPRH also directly ubiquitinates unmodified PCNA at Lys164. The reaction requires the Ube2D family of ubiquitin conjugating enzymes and robustly ubiquitinates both free and DNA-bound PCNA. We further found that efficient PCNA ubiquitination requires SHPRH’s HIRAN domain, which mediate interactions with PCNA. Our data suggest another layer of regulation of DNA damage responses through PCNA ubiquitination.

## Introduction

Multiple stressing factors, including DNA lesions, nucleotide limitation, transcription complexes and others, can stall DNA replication. If left unresolved, stalled DNA replication can lead to genomic instability and/or cell death [1,2]. The DNA damage tolerance (DDT) pathway plays a critical role in overcoming the replication stresses. DDT responses are primarily regulated by the ubiquitination of the proliferating cell nuclear antigen (PCNA), which forms a trimeric ring-like structure that encircles DNA during replication, acting as a binding platform for polymerases and other replication factors [3,4]. In the budding yeast, PCNA mono-ubiquitination by the Rad6-Rad18 complex at a highly conserved Lys164 residue signals for translesion synthesis (TLS). The mono-ubiquitinated PCNA recruits the Y-family of DNA polymerases, which can catalyze replication across template lesions, but could introduce errors. Mono-ubiquitinated PCNA can be further poly-ubiquitinated by the RING family of ubiquitin ligase (E3) Rad5 together with the ubiquitin-conjugating complex (E2) Ubc13-Mms2, which attaches lysine-63-linked ubiquitin chain to Lys164 in PCNA. Such PCNA polyubiquitination signals for template-switch recombination, initiating the error-free branch of DDT [5–8].

The DDT pathway is highly conserved in eukaryotes. In human cells, Rad5 orthologues SNF2 histone linker PHD RING helicase (SHPRH) and helicase like transcription factor (HLTF) together with the human Ubc13-MmS2 complex catalyze PCNA polyubiquitination, following its mono-ubiquitination by the human Rad18-Rad6 complex [9–13]. The Rad5 family of enzymes are multiple domain proteins. In addition to the RING domain typical for the RING family of E3 enzymes, they also contain a Snf2 family of DNA translocase domain [14]. It has been reported that the Snf2 domain in Rad5 and HLTF drive replication fork regression, which may protect the stalled the replication fork and/or provide template for recombination [15–18]. Rad5 and HLTF also contain a HIRAN domain at their N-terminus. HLTF’s HIRAN domain has been shown to bind the DNA 3’-OH group and plays a critical role in the HLTF-catalyzed replication fork regression [19–21]. Similarly, we have recently found that Rad5’s HIRAN domain also mediates interactions with DNA and is critical for the Rad5-catalyzed replication fork regression, although it does not recognize the DNA 3’-OH group. Interestingly, we found that Rad5’s HIRAN domain also mediates interactions with PCNA, playing a critical role in the Rad5-catalyzed PCNA ubiquitination [14,15]. Structure-guided sequence analysis suggested that SHPRH may also contain a HIRAN domain in its N-terminal region [22], but its function is not clear. Compared to Rad5 and HLTF, SHPRH contains additional linker histone H15 and PHD domains in its N-terminal region. The PHD domain recognizes distinct methylation states on histone H3, enabling precise recruitment of SHPRH for DNA damage repair [23,24].

In addition to the canonical PCNA poly-ubiquitination reaction described above, Rad5 has also been reported to directly ubiquitinate unmodified PCNA with the E2 enzyme Ubc4 [25]. We have recently reported that the *K. lactis* Rad5 together with Ubc4 ubiquitinates PCNA at Lys164 *in vitro* [26]. Such reaction adds another layer of complexity to the regulation of DDT responses. To date, it is not clear if the human Rad5 orthologs catalyze a similar reaction.

Here, using purified proteins, we show that SHPRH together with the Ube2D family of E2 enzymes directly and robustly ubiquitinates unmodified PCNA *in vitro*. We found that the SHPRH and Ube2D ubiquitinate both free PCNA and DNA-bound PCNA and efficient ubiquitination requires SHPRH’s HIRAN domain. Importantly, we found that the reaction primarily modifies Lys164 in PCNA, suggesting that it may also play a role in regulating DDT responses.

## Materials and methods

### Proteins

The human Uba1 (isoform 1, UniProt code P22314) was expressed in *Spodoptera frugiperda* (Sf9) cells and purified with nickel-nitrilotriacetic acid (Ni-NTA, Smart-Lifesciences) and size-exclusion (Superdex 200 10/300, GE Healthcare) columns as previously described [27]. The human SHPRH (isoform X1, UniProt code A0A0D9SFM0) gene was codon-optimized for expression in bacteria, synthesized (Tsingke Biotechnology, S1 Table) and inserted into the vector pMAL-c2X (New England Biolabs). SHPRH with an N-terminal maltose-binding protein (MBP) tag was expressed in *Escherichia coli* BL21 Rosetta (DE3) cells and purified by dextrin resin (Smart-Lifesciences), ion-exchange (Hitrap Heparin HP, Cytiva), and size-exclusion (Superdex 200 increase 10/300, Cytiva) columns. The synthesized gene fragment coding SHPRH’s HIRAN domain (residues 2–270) was inserted into vector pET26B (Novagen). The recombinant SHPRH’s HIRAN domain with a C-terminal 6x histidine tag was expressed in BL21 Rosetta (DE3) cells and purified by Ni-NTA, ion-exchange (Hitrap SP HP, Cytiva), and size-exclusion (Superdex 75 increase 10/300, Cytiva) columns. Ube2D1/2/3/4 (isoform 1, UniProt codes P51668, P62837, P61077 and Q9Y2X8) genes were inserted into the vector pET28A (Novagen). Ube2D1/2/3/4 with an N-terminal 6x histidine tag were expressed in BL21 Rosetta (DE3) cells and purified with Ni-NTA and size-exclusion (Superdex 200 increase 10/300) columns. To purify the HA-tagged PCNA (isoform 1, UniProt code P12004), the PCNA gene was inserted into vector pET28A, with an oligonucleotide encoding the hemagglutinin (HA) tag attached to its 5’ end. PCNA with N-terminal 6x histidine and HA tags were expressed in BL21 Rosetta (DE3) cells and purified by Ni-NTA, ion-exchange (Hitrap Q HP) and size-exclusion (Superdex 200 increase 10/300) columns. To purify the strep-tagged PCNA, the PCNA gene was inserted into vector pET26B together with an oligonucleotide encoding the strep-tag. N-terminally strep-tagged and C-terminally 6x histidine-tagged PCNA was expressed in BL21 Rosetta (DE3) cells and purified following the same method for HA-PCNA. The ubiquitin gene was inserted into vector pET28A. Ubiquitin with an N-terminal 6x histidine tag was expressed in BL21 Rosetta (DE3) cells and purified with Ni-NTA and size-exclusion (Superdex 75 increase 10/300) columns. The expression and purification methods of the human RFC complex was adopted from a previous study [28]. Briefly, the RFC1 (isoform 1, UniProt code P35251) gene was inserted into the vector pCDFDuet (Novagen), the RFC2, RFC3, RFC4 and RFC5 (isoform 1, UniProt codes P35250, P40938, P35249 and P40937) genes were inserted into the vector pETDuet (Novagen). The above two plasmids were co-transformed into BL21 (DE3) cells to express the RFC complex, which was purified by ion-exchange (Hitrap SP HP), Ni-NTA and size-exclusion (Superdex 6 increase 10/300) columns. The human Rad6a (isoform 1, UniProt code P49459) gene was inserted into vector pTXb1. Rad6a with a C-terminal intein tag was expressed in BL21 Rosetta (DE3) cells and purified by chitin resin, ion-exchange (Hitrap Q HP, Cytiva), and size-exclusion (Superdex 75 increase 10/300, Cytiva) columns. The human Rad18 (isoform 1, UniProt code Q9NS91) gene was inserted into vector pET28A together with 6x histidine tag and strep-tag. N-terminally strep-tagged and 6x histidine-tagged Rad18 was expressed in BL21 Rosetta (DE3) cells and purified by strep-tactin beads, ion-exchange (Hitrap Heparin HP, Cytiva), and size-exclusion (Superdex 200 increase 10/300, Cytiva) columns. Amino acid substitutions and deletions were generated by polymerase chain reactions following instructions of the QuikChange kit (Agilent Technologies) and verified by DNA sequencing. The expression and purification of the substituted proteins followed the same method for the wild type proteins.

### *In vitro* PCNA ubiquitination by SHPRH and Ube2D1/2/3/4

The ubiquitination reaction mixture for free PCNA contains 40 mM Tris-HCl (pH 7.5), 50 mM sodium chloride, 10 mM magnesium chloride, 1 mM ATP, 0.05 μM Uba1, 20 μM Ube2D1/2/3/4, 2.5 μM SHPRH, 30 μM ubiquitin, and 0.025 μM PCNA. The reactions were allowed to proceed at 30 ºC for the indicated durations and terminated by boiling in SDS-PAGE loading buffer.

DNA-bound PCNA was prepared as described [29]. Briefly, a circular DNA containing multiple single- and double-stranded regions were prepared by annealing multiple oligonucleotides to a circular single-stranded DNA. One of the oligonucleotides contains a biotin tag. To assemble the PCNA-DNA complex, 1 μg of this DNA molecule was incubated with 25 μL of strep-tactin beads equilibrated in reaction buffer (20 mM Tris pH 7.5, 50 mM sodium chloride, 10 mM magnesium chloride, 1 mM dithiothreitol (DTT), 0.2 mg/mL bovine serum albumin) for 1 hour at 4 ºC. The beads were washed three times with 1 mL of the reaction buffer, and incubated with 15 μM PCNA, 0.6 μM RFC, and 1 mM ATP for 10 minutes at 30 ºC. Unbound proteins were removed by washing the beads six times with the reaction buffer. The ubiquitination reaction for the DNA-bound PCNA complex was identical to that for free PCNA, except that the free PCNA was replaced by the PCNA-DNA complex-bound strep-tactin beads (25 μL beads per 100 μL reaction), and the reaction mixture was supplemented with 0.2 mg/mL BSA and 1 mM DTT. Reactions were allowed to proceed at 30 ºC for the indicated durations and were terminated by boiling in SDS-PAGE loading buffer.

The reactions were analyzed with Western blotting with rabbit anti-HA (C29F4, Cell Signaling Technology, 1:2000 diluted) and horseradish peroxidase–conjugated goat anti-rabbit IgG (ZB-2301, ZSGB-BIO, 1:10000 diluted) antibodies.

### *In vitro* PCNA ubiquitination by Rad18 and Rad6

The Rad18-Rad6 catalyzed PCNA ubiquitination reactions were carried out following a previously published method with some modifications [30]. The reaction mixture contains 40 mM Tris-HCl (pH 7.5), 50 mM sodium chloride, 10 mM magnesium chloride, 1 mM ATP, 0.2 mg/mL BSA, 1 mM DTT, 0.05 μM Uba1, 3 μM Rad6a, 1 μM Rad18, 30 μM ubiquitin, and strep-tactin beads bound with the DNA-PCNA complex (25 μL beads per 100 μL reaction). Reactions were allowed to proceed at 30 °C for the indicated time, terminated by boiling in SDS-PAGE loading buffer, and analyzed with western blotting as described above.

### Pull-down assay

Purified strep-PCNA (10 μg) was incubated with 50 uL strep-tactin beads equilibrated in buffer A (20 mM Tris (pH7.5), 200 mM sodium chloride, 1 mM DTT) for 1 hour at 4 ºC. After washing the beads five times with buffer A, they were further incubated with 10 μg SHPRH’s HIRAN domain for 1 hour at 4 ºC in buffer A. Beads were again washed five times with buffer A, and bound proteins were eluted with buffer A containing 10 mM D-Desthiobiotin. Samples were analyzed with Western blotting with the HRP conjugated mouse anti-Histag antibody (ab5000, Abcam, 1:1000 diluted) or SDS-PAGE.

## Results

### SHPRH and the Ube2D family of E2 enzymes directly ubiquitinate free PCNA

**Fig 1:**
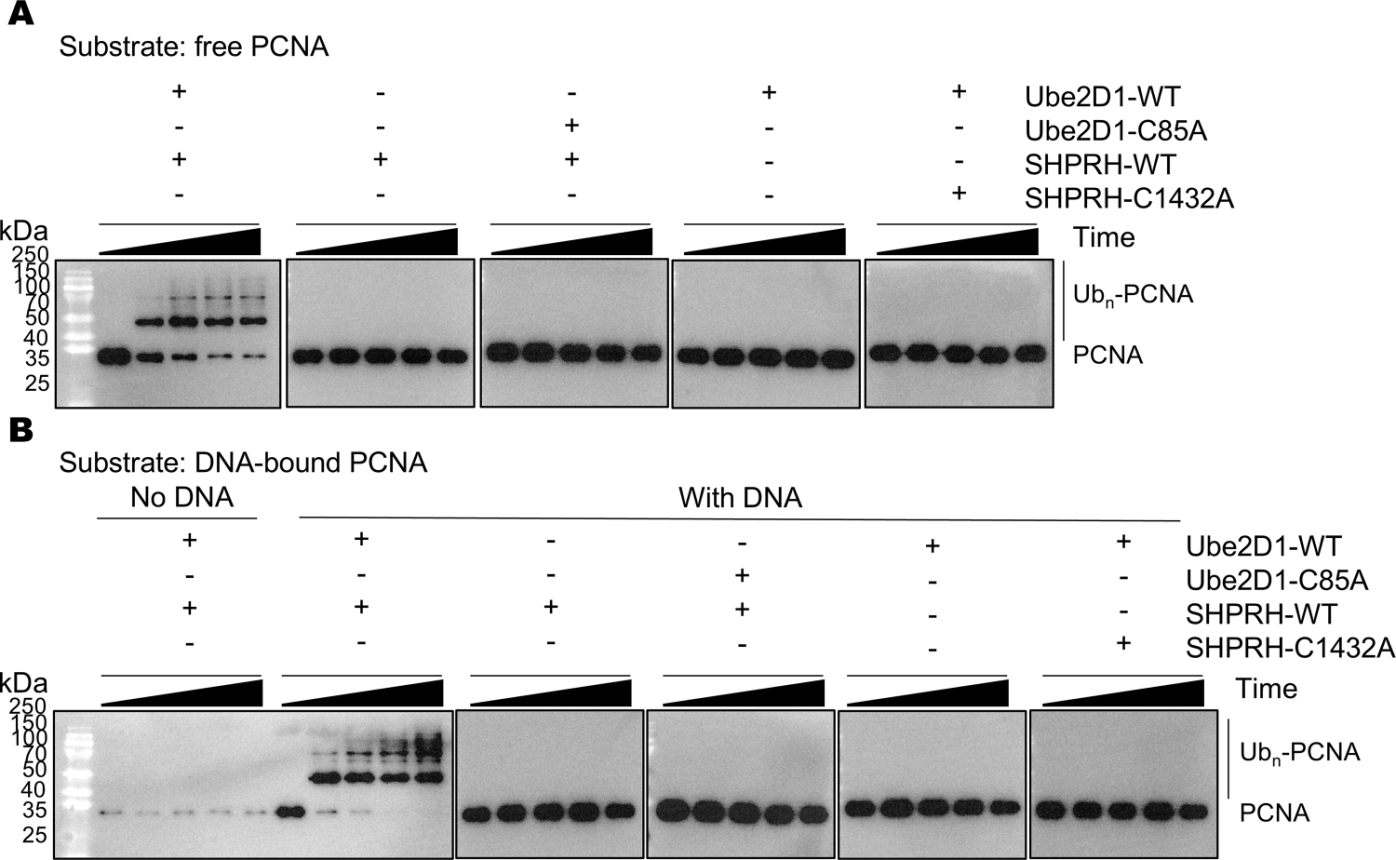
SHPRH and Ube2D1 directly ubiquitinate PCNA. **(A)** SHPRH and Ube2D1 ubiquitinate free PCNA. **(B)** SHPRH and Ube2D1 ubiquitinate DNA-bound PCNA. Western blot analyses for PCNA are presented. The reactions were allowed to proceed for 0, 5, 10, 20, and 40 minutes before termination.

Among the human E2 enzymes, members in the Ube2D family are most homologous to Ubc4 in the budding yeast. Ube2D1-4 share ~80% amino acid sequence identity with the budding yeast Ubc4. We tested if SHPRH together with the Ube2D family of E2 enzymes could ubiquitinate PCNA with purified SHPRH, Ube2D1-4 and related proteins (S1 Fig). We found that SHPRH together with Ube2D1/2/3/4 robustly ubiquitinates unbound PCNA (Fig 1A and S2A-4A Figs). A predominate modified PCNA species of ~50 kDa is produced. Comparing with the reaction by the Rad6-Rad18 complex that mono-ubiquitinates PCNA (S5 Fig) suggests that it is mono-ubiquitinated PCNA. Additional larger molecular weight bands presumably represent poly-ubiquitinated PCNA. Therefore, the reaction produces both mono- and poly-ubiquitinated PCNA.

Despite extensive optimization, the purified SHPRH used in the above experiments is only about 50% pure (S1 Fig). SHPRH is purified by dextrin resin that binds to a maltose-binding protein (MBP) tag fused to its N-terminus, followed by ion-exchange and size-exclusion columns. The dextrin resin-MBP binding is highly specific, suggesting that the impurities are mostly due to SHPRH protein degradation. Indeed, SHPRH is predicted to contain multiple flexible regions that are susceptible to proteolysis cleavage. To rule out the possibility that the observed PCNA ubiquitination is catalyzed by contaminating proteins from the expression host, we purified the C1432A-substitued SHPRH and tested its ability in ubiquitinating PCNA. The C1432A substitution into SHPRH’s RING domain abolishes its E3 activity [12]. We found that the C1432A-substitued SHPRH was unable to ubiquitinate PCNA (Fig 1A and S3A-5A Figs), suggesting that the observed PCNA ubiquitination is catalyzed by SHPRH but not contaminating proteins. Similarly, the C85A substitution into the active site of Ube2D1-4 also abolished ubiquitination of free PCNA (Fig 1A and S2A-4A Figs). Together, these data indicate that SHPRH and the Ube2D family of E2 enzymes directly and efficiently ubiquitinates free PCNA *in vitro*.

### SHPRH and the Ube2D family of E2 enzymes also ubiquitinate DNA-bound PCNA

During DNA replication, PCNA is loaded onto DNA. Loading PCNA onto DNA is required for its ubiquitination by the Rad6-Rad18 complex [30,31]. Compared to free PCNA, the altered molecular surface of the DNA-PCNA complex may enable the specific recognition by the Rad6-Rad18 complex. We next tested whether SHPRH and Ube2D1-4 can also recognize the DNA-PCNA complex and ubiquitinate DNA-bound PCNA. We purified DNA-bound PCNA following a previously published method [29]. Briefly, PCNA was loaded onto a circular DNA molecule containing multiple single-stranded and double-stranded regions with the replication factor C (RFC) complex (Fig S1), and the DNA-PCNA complex was purified with strep-tactin resin that binds to the DNA molecule containing a biotin tag. Little PCNA was detected in a mock experiment without DNA (Fig. 1B and S2B-4B Figs), suggesting that the majority of the purified PCNA was DNA-bound. We found that the DNA-bound PCNA was also robustly ubiquitinated by SHPRH and Ube2D1/2/3/4, and PCNA ubiquitination was strongly inhibited by the C1432A substitution in SHPRH or the C85A substitution in Ube2D1-4 (Fig 1B and S3B-5B Figs). Together, these data indicate that SHPRH and Ube2D1-4 also efficiently ubiquitinate DNA-bound PCNA.

### SHPRH’s HIRAN domain mediates interactions with PCNA

**Fig 2:**
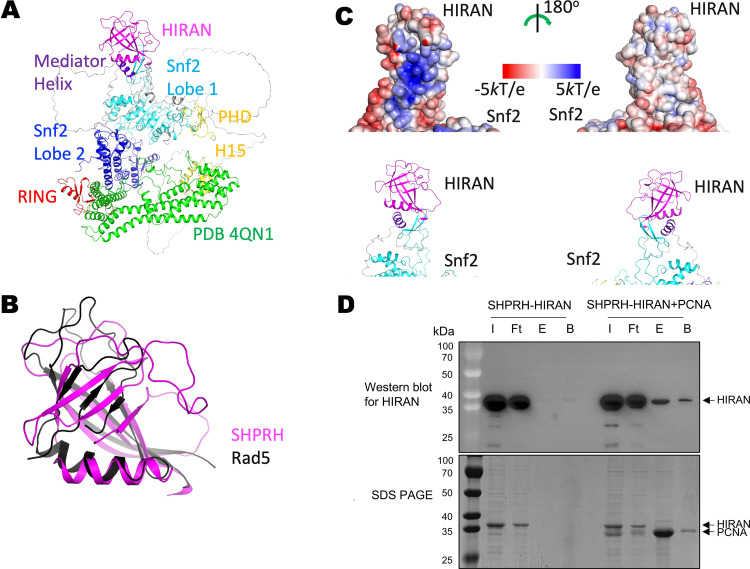
SHPRH’s HIRAN domain mediates interactions with PCNA. **(A)** The Alphafold-predicted structure of SHPRH. SHPRH domains are represented in different colors. The region colored in green indicates a SHPRH fragment with an experimentally determined structure (PDB 4QN1). The function of this fragment is unknown. **(B)** Structural comparison of HIRAN domains in SHPRH and Rad5. **(C)** Electrostatic potential on SHPRH’s HIRAN domain. The surface of SHPRH is colored according to the electrostatic potential. In the lower panels, ribbon representations of SHPRH in identical orientations are shown for reference. **(D)** Pulldown experiments probing the interaction between PCNA and SHPRH’s HIRAN domain. Western blot (for the HIRAN domain, upper panel) and SDS-PAGE (lower panel) analyses of the samples are presented. I, input protein; Ft, unbound (flowthrough) fraction; E, eluted fraction; B, the strep-tactin beads after elution.

In line with previous sequence analysis [22], structure prediction by AlphaFold and recent cryo-EM structure of SHPRH [32] indicated that it contains an N-terminal HIRAN domain that is homologous to the HIRAN domain in Rad5 and HLTF (Figs 2A-2B). Rad5’s HIRAN domain contains a positively charged surface patch that mediate interactions with PCNA [14,15]. Similarly, SHPRH’s HIRAN domain also contains a predominantly positively charged surface patch, although its location is slightly different from the positively charged surface patch in Rad5’s HIRAN domain (Fig 2C). To test if SHPRH’s HIRAN domain also mediate interactions with PCNA, we purified it and characterized its binding with PCNA with a pulldown experiment. We found that SHPRH’s HIRAN domain binds to strep-tactin resin pre-loaded with strep-tagged PCNA but not to the step-tactin resin alone (Fig 2D). Therefore, like Rad5’s HIRAN domain, SHPRH’s HIRAN domain also mediate direct interactions with PCNA.

### SHPRH’s HIRAN domain is required for efficient PCNA ubiquitination

**Fig 3:**
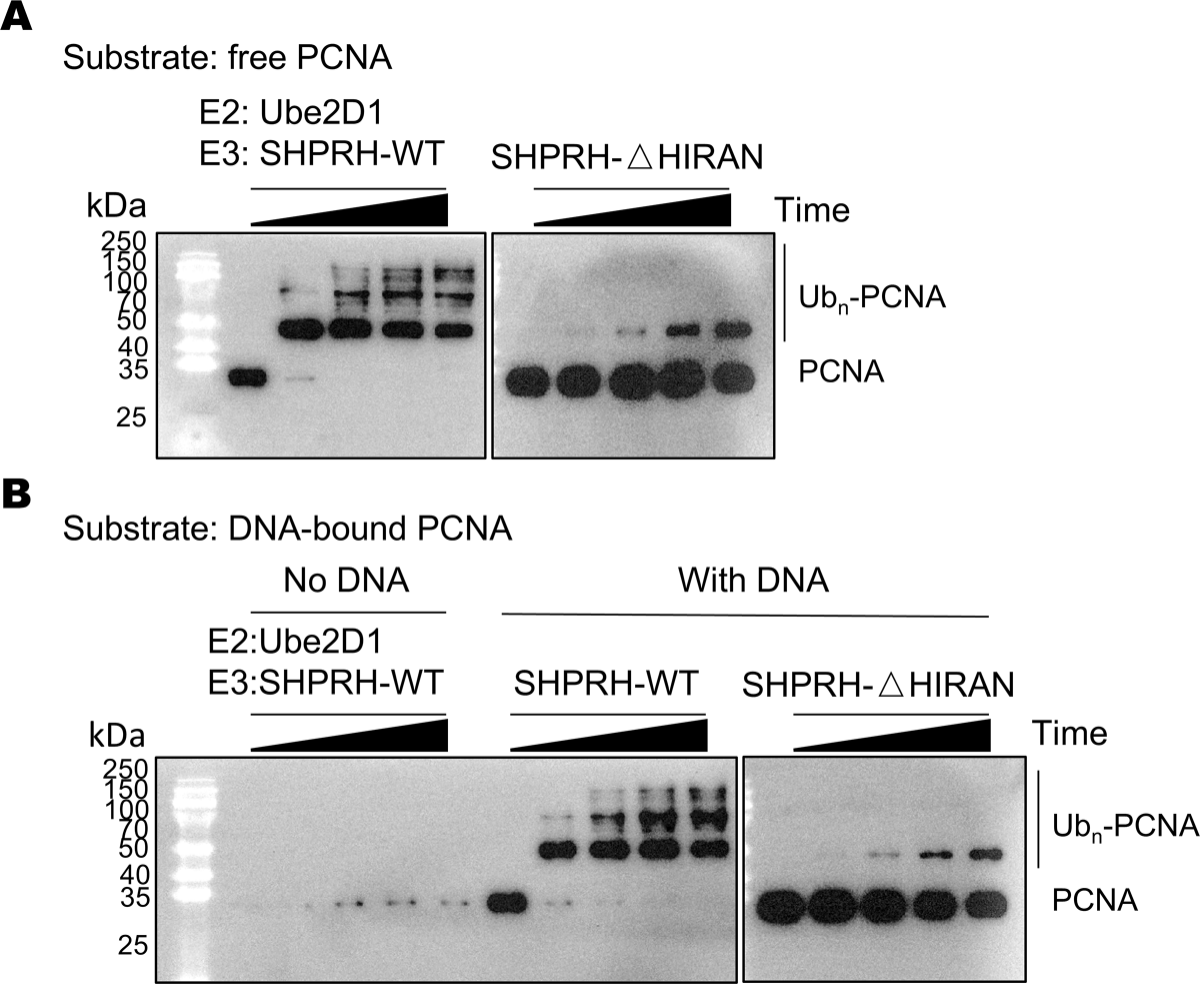
SHPRH’s HIRAN domain is required for efficient PCNA ubiquitination. Western blot analysis of ubiquitination of free PCNA (A) and DNA-bound PCNA (B) by the SHPRH-△HIRAN and Ube2D1 is presented. Reactions with the wild type SHPRH were included for comparison. The reactions were allowed to proceed for 0, 5, 10, 20, and 40 minutes before termination.

We have recently reported that the HIRAN-PCNA interaction is important for the Rad5-catalyzed PCNA ubiquitination reaction [14,15]. To tested if it also contributes to the SHPRH and Ube2D1/2/3/4-catalyzed PCNA ubiquitination, we purified a SHPRH variant lacking the HIRAN domain (SHPRH-△HIRAN, residues 1–251 removed, S1 Fig) and probed its activity to ubiquitinate PCNA with Ube2D1/2/3/4 (Fig 3 and S6-8 Figs). We found that this SHPRH variant together with Ube2D1/2/3/4 can still ubiquitinate unbound PCNA and DNA-bound PCNA, but the activity is significantly lower than the wild type SHPRH. Therefore, the HIRAN domain is required for efficient PCNA ubiquitination by SHPRH and Ube2D1//2/3/4.

### SHPRH and the Ube2D family of E2 enzymes primarily ubiquitinate PCNA at the conserved Lys164

**Fig 4:**
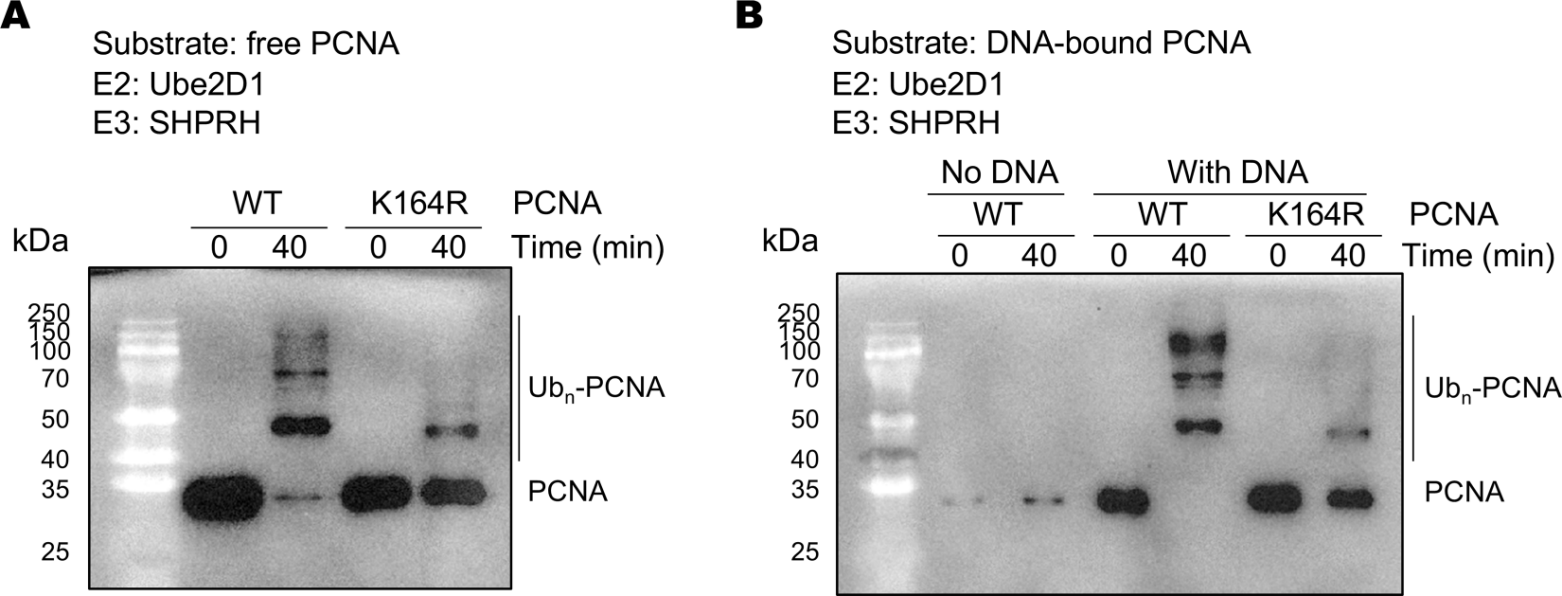
SHPRH and Ube2D1 primarily ubiquitinate PCNA at the conserved Lys164. Western blot analysis of ubiquitination reactions with free PCNA (A) and DNA-bound PCNA (B) by SHPRH and Ube2D1 is presented. Reactions with the wild type and K164R-substituted PCNA are presented.

The highly conserved Lys164 site in PCNA is subject to ubiquitination and sumolyation modifications [33]. We have recently reported that the *K. lactis* Rad5 together with Ubc4 ubiquitinates PCNA at Lys164 [26]. To test if SHPRH and Ube2D1-4 also ubiquitinate PCNA at this site, we repeated the ubiquitination reactions with the K164R-subsituted PCNA. We found in both reactions with free PCNA and DNA-bound PCNA, PCNA ubiquitination is severely inhibited by the K164R substitution (Fig 4 and S9-11 Figs). These data indicates that SHPRH and Ube2D1-4 primarily ubiquitinates PCNA at the highly conserved Lys164 site. Weak PCNA ubiquitination signals can be observed in reactions with the K164R-subsituted PCNA, suggesting for additional, minor ubiquitination sites. Similar observations have been made on the PCNA ubiquitination reaction by *K. lactis* Rad5 and Ubc4 [26].

## Discussion

We report here that SHPRH together with the Ube2D family of E2 enzymes efficiently ubiquitinates both free and DNA-bound PCNA *in vitro*, and the highly conserved Lys164 is the primary site of ubiquitination. Our data suggests for additional, minor ubiquitination sites. Hence, it remains to be seen whether the observed poly-ubiquitination signal arises from poly-ubiquitination of Lys164, or mono-ubiquitination of multiple sites in PCNA. In contrast to the canonical PCNA ubiquitination by SHPRH and the Ubc13-Mms2 complex, which requires mono-ubiquitinated PCNA, we found that SHPRH and Ube2D1-4 directly ubiquitinates unmodified PCNA. Post-translational modifications on PCNA Lys164 play critical roles in DNA damage or replication stress responses [34]. In line with this function, recent studies on cells harboring the unmodifiable K164R-substituted PCNA revealed multiple defects, including global DNA replication disorders, cell cycle arrest, increased sensitivity towards DNA damage agents, inhibited TLS, and others [35,36]. Previous studies indicated that this site can be sumolylated, ubiquitinated by Rad18 and Rad6, Rad5/SHPRH/HLTF and the Ubc13-Mms2 complex, or Ubc4 [5,11,13,26,37]. It remains to be seen whether the PCNA ubiquitination reaction by SHPRH and Ube2D1-4 takes place *in vivo*. If so, it may contribute to the complex regulation of DNA damage/replication stress responses through yet another mechanism.

The HIRAN domain is a conserved feature of the Rad5 family of enzymes, yet previous studies have identified distinct functions of this domain in HLTF and Rad5. HLTF’s HIRAN domain has been found to mediate specific interactions with the DNA 3’-OH group and play a critical role in the HLTF-catalyzed replication fork regression [19–21]. Rad5’s HIRAN domain also mediates interactions with DNA and is required for efficient fork regression by Rad5, but it does not recognize the 3’-OH group. In addition, it also mediates interactions with PCNA and is required for the Rad5-catalyzed PCNA ubiquitination [14,15]. Here we found that SHPRH’s HIRAN domain also mediate interaction with PCNA is required for efficient PCNA ubiquitination by SHPRH and the Ube2D family of E2 enzymes. It would be interesting to study whether the HIRAN domain also plays a role in the canonical PCNA ubiquitination reaction by SHPRH and the Ubc13-Mms2 complex.

In summary, we provide *in vitro* evidence that SHPRH and the Ube2D family of E2 enzymes directly ubiquitinate PCNA primarily at Lys164, and efficient reaction requires SHPRH’s HIRAN domain that interacts with PCNA. Our data suggests another layer of regulation of DNA damage responses through PCNA ubiquitination and expands the understanding of HIRAN domains’ function in the Rad5 family of enzymes.

## Supporting information

S1 Fig Proteins and the RFC complex used in this study. SDS PAGE analysis of the purified proteins and the RFC complex is presented. The corresponding protein bands are indicated by the red arrowheads.
(TIF)

S2 Fig SHPRH and Ube2D2 directly ubiquitinate free PCNA (A) and DNA-bound PCNA (B). The reactions are the same as those presented in figure 1, except that the E2 enzyme is Ube2D2.
(TIF)

S3 Fig SHPRH and Ube2D3 directly ubiquitinate free PCNA (A) and DNA- bound PCNA (B). The reactions are the same as those presented in figure 1, except that the E2 enzyme is Ube2D3.
(TIF)

S4 Fig SHPRH and Ube2D4 directly ubiquitinate free PCNA (A) and DNA- bound PCNA (B). The reactions are the same as those presented in figure 1, except that the E2 enzyme is Ube2D4.
(TIF)

S5 Fig PCNA mono-ubiquitination reaction catalyzed by Rad6 and Rad18. Western blot analysis for PCNA after the reaction are presented. The reactions were allowed to proceed for 0, 5, 10, 20, and 40 minutes before analysis.
(TIF)

S6 Fig SHPRH’s HIRAN domain is required for efficient PCNA ubiquitination reaction. Western blot analysis of ubiquitination of free PCNA (A) and DNA-bound PCNA (B) by the wild type SHPRH or SHPRH-△HIRAN and Ube2D2 is presented. The reactions are the same as reactions presented in figure 3, except that the E2 enzyme is Ube2D2.
(TIF)

S7 Fig SHPRH’s HIRAN domain is required for efficient PCNA ubiquitination reaction. Western blot analysis of ubiquitination of free PCNA (A) and DNA-bound PCNA (B) by the wild type SHPRH or SHPRH-△HIRAN and Ube2D3 is presented. The reactions are the same as reactions presented in Fig 3, except that the E2 enzyme is Ube2D3.
(TIF)

S8 Fig SHPRH’s HIRAN domain is required for efficient PCNA ubiquitination reaction. Western blot analysis of ubiquitination of free PCNA (A) and DNA-bound PCNA (B) by the wild type SHPRH or SHPRH-△HIRAN and Ube2D4 is presented. The reactions are the same as reactions presented in figure 3, except that the E2 enzyme is Ube2D4.
(TIF)

S9 Fig SHPRH and Ube2D2 primarily ubiquitinate PCNA at the conserved Lys164. Reactions for free PCNA (A) and DNA-bound PCNA(B) are presented. The reactions are the same as those presented in figure 4, except that the E2 enzyme is Ube2D2.
(TIF)

S10 Fig SHPRH and Ube2D3 primarily ubiquitinate PCNA at the conserved Lys164. Reactions for free PCNA (A) and DNA-bound PCNA(B) are presented. The reactions are the same as those presented in figure 4, except that the E2 enzyme is Ube2D3.
(TIF)

S11 Fig SHPRH and Ube2D4 primarily ubiquitinate PCNA at the conserved Lys164. Reactions for free PCNA (A) and DNA-bound PCNA(B) are presented. The reactions are the same as those presented in figure 4, except that the E2 enzyme is Ube2D4.
(TIF)

S1 Table Sequence of the synthesized SHPRH gene. (PDF)
