# Supplementary figures and images for: SHPRH together with the Ube2D family of enzymes directly ubiquitinates PCNA at Lys164 in vitro
Source: PLoS One. 2026 Apr 16;21(4):e0347227. doi: 10.1371/journal.pone.0347227 (PMC13086300; doi:10.1371/journal.pone.0347227)

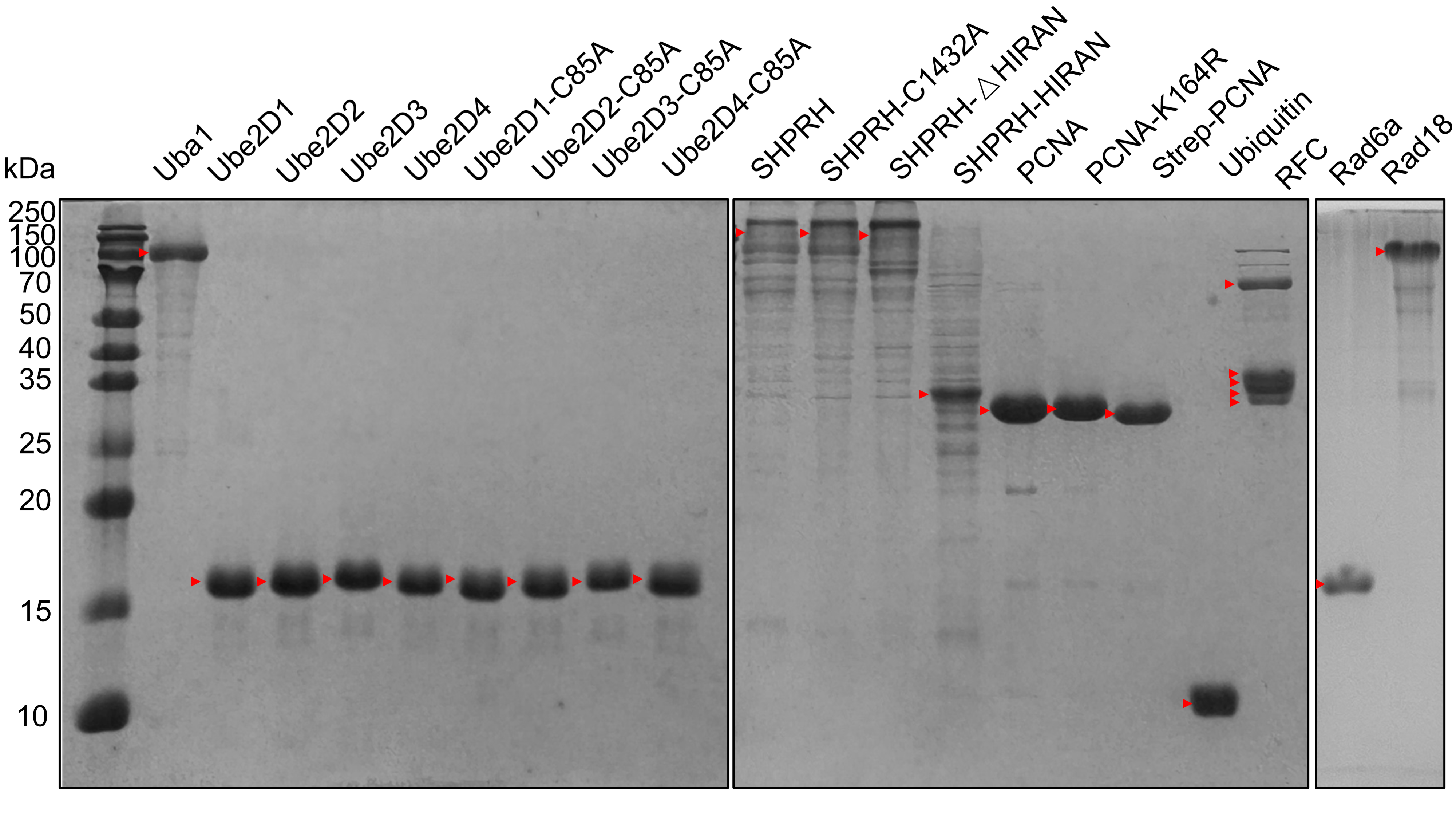

Supplement: S1 Fig — SDS PAGE analysis of the purified proteins and the RFC complex is presented. The corresponding protein bands are indicated by the red arrowheads. (TIF) [file pone.0347227.s001.tif]

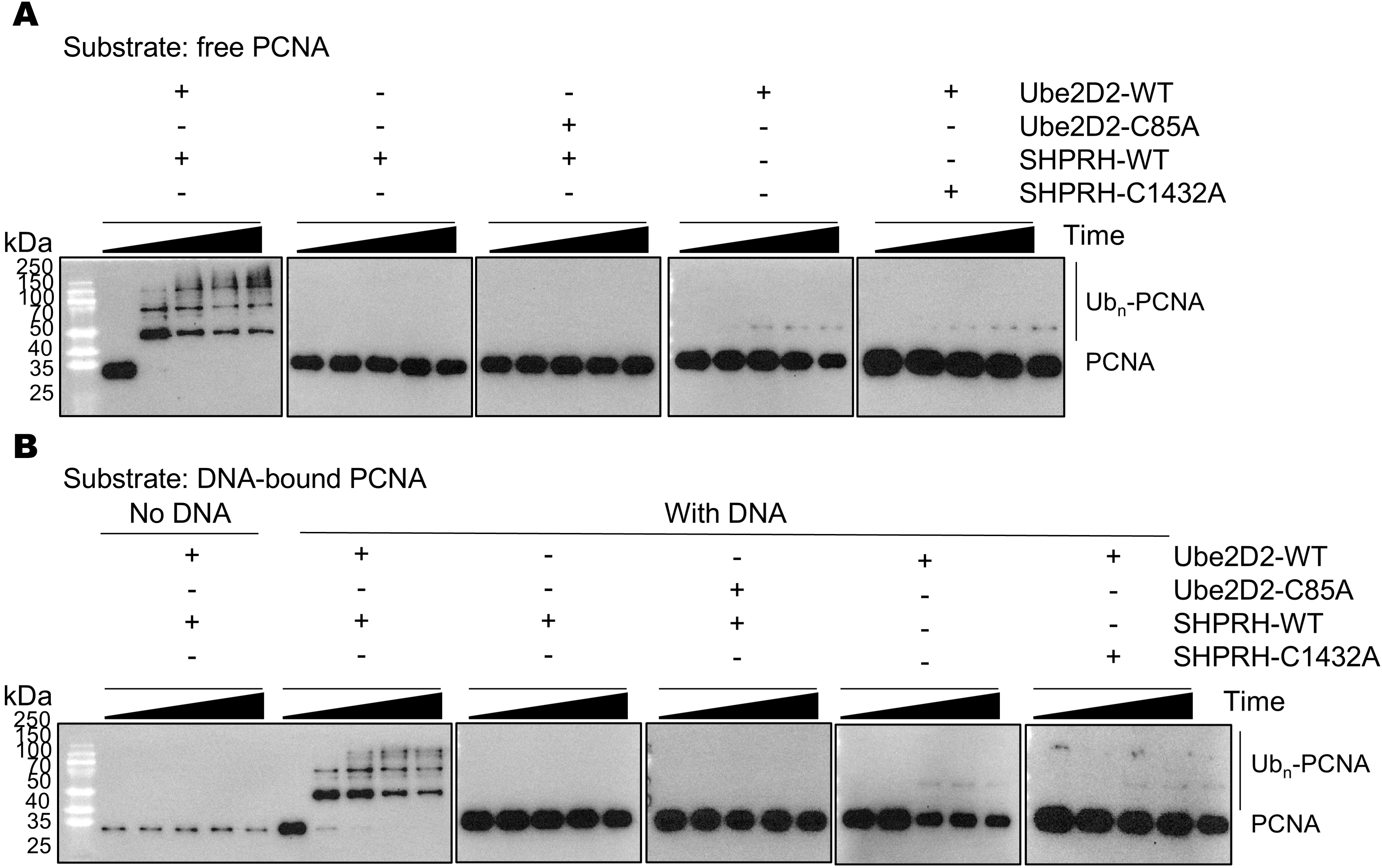

Supplement: S2 Fig — The reactions are the same as those presented in figure 1, except that the E2 enzyme is Ube2D2. (TIF) [file pone.0347227.s002.tif]

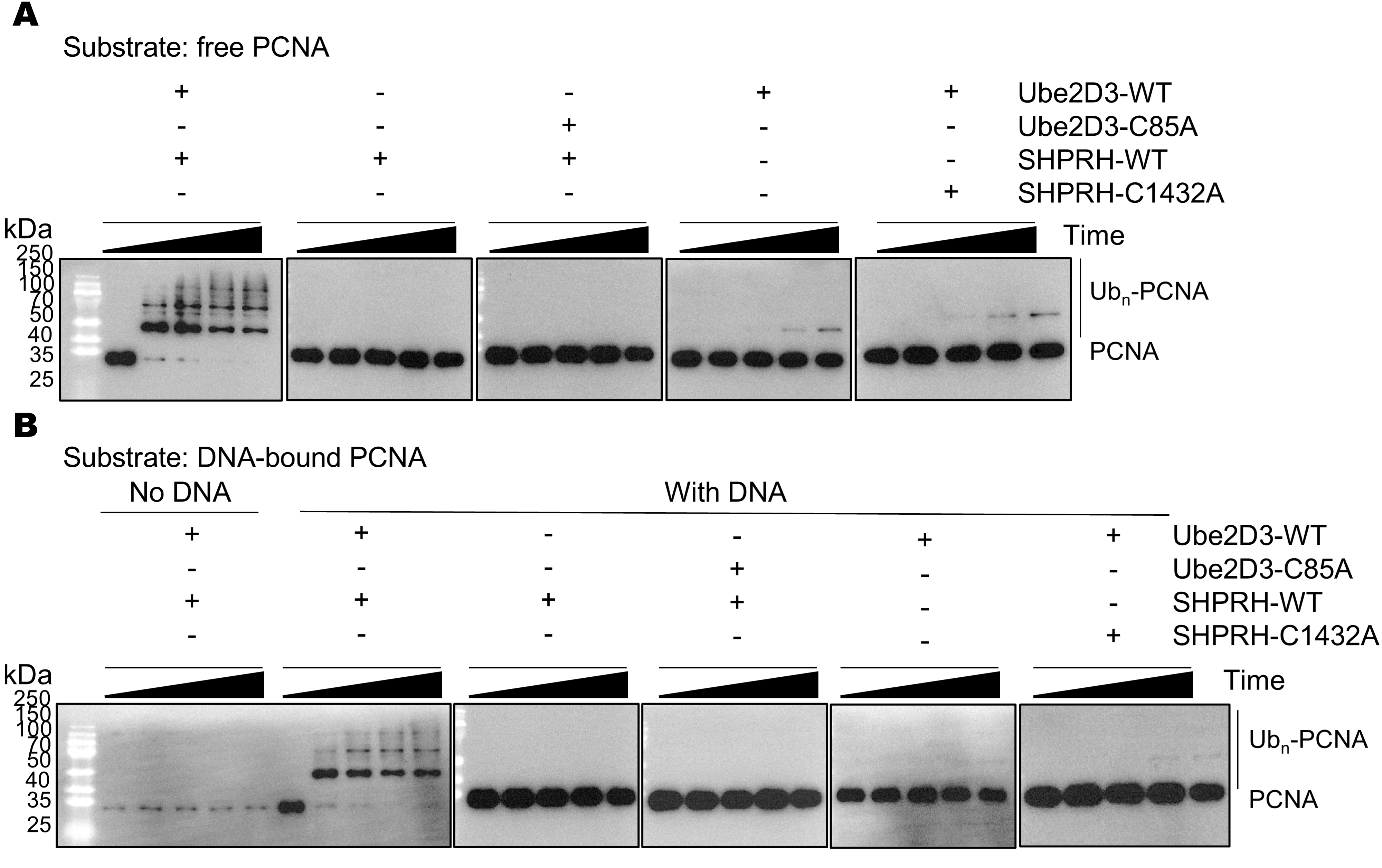

Supplement: S3 Fig — The reactions are the same as those presented in figure 1, except that the E2 enzyme is Ube2D3. (TIF) [file pone.0347227.s003.tif]

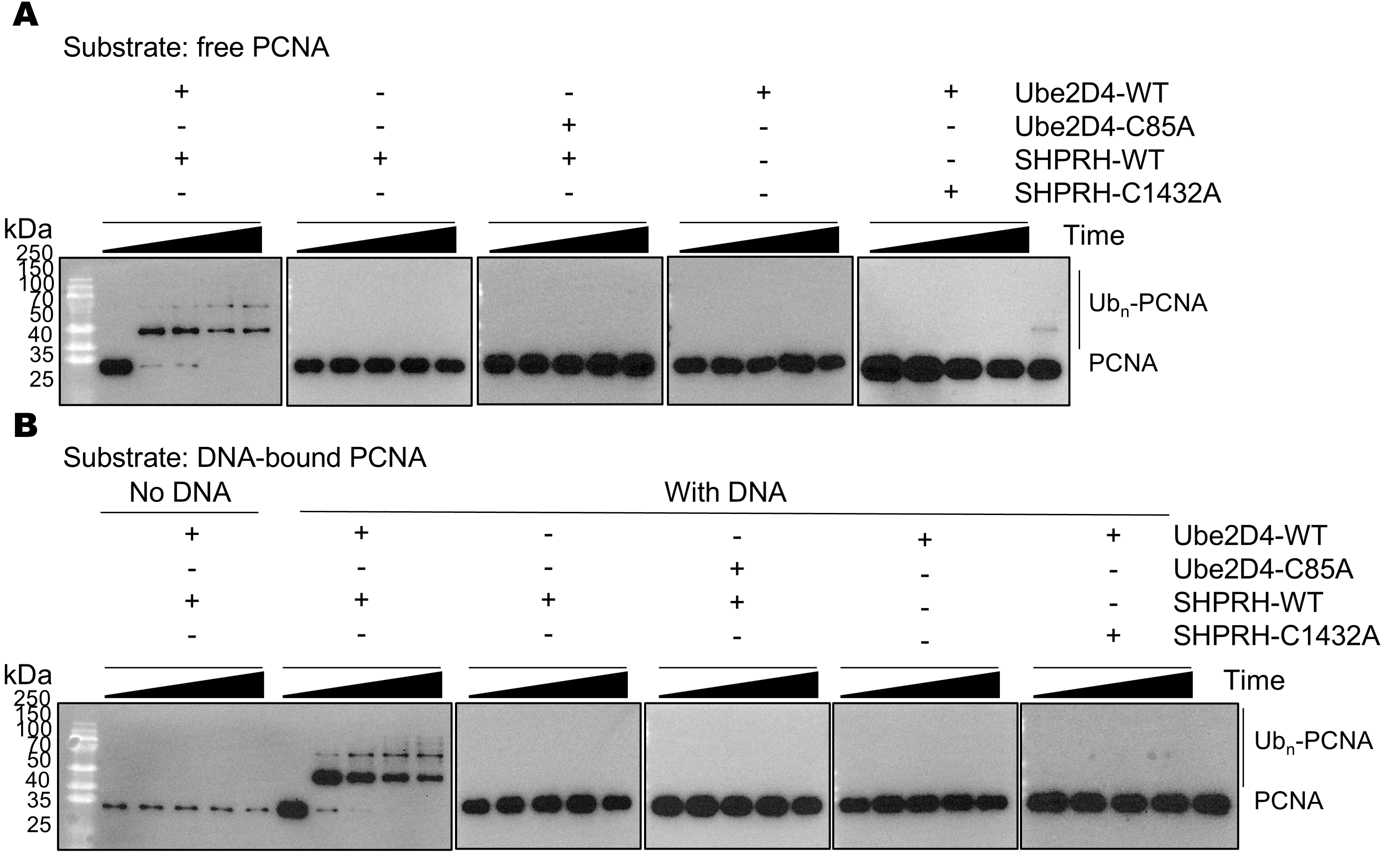

Supplement: S4 Fig — The reactions are the same as those presented in figure 1, except that the E2 enzyme is Ube2D4. (TIF) [file pone.0347227.s004.tif]

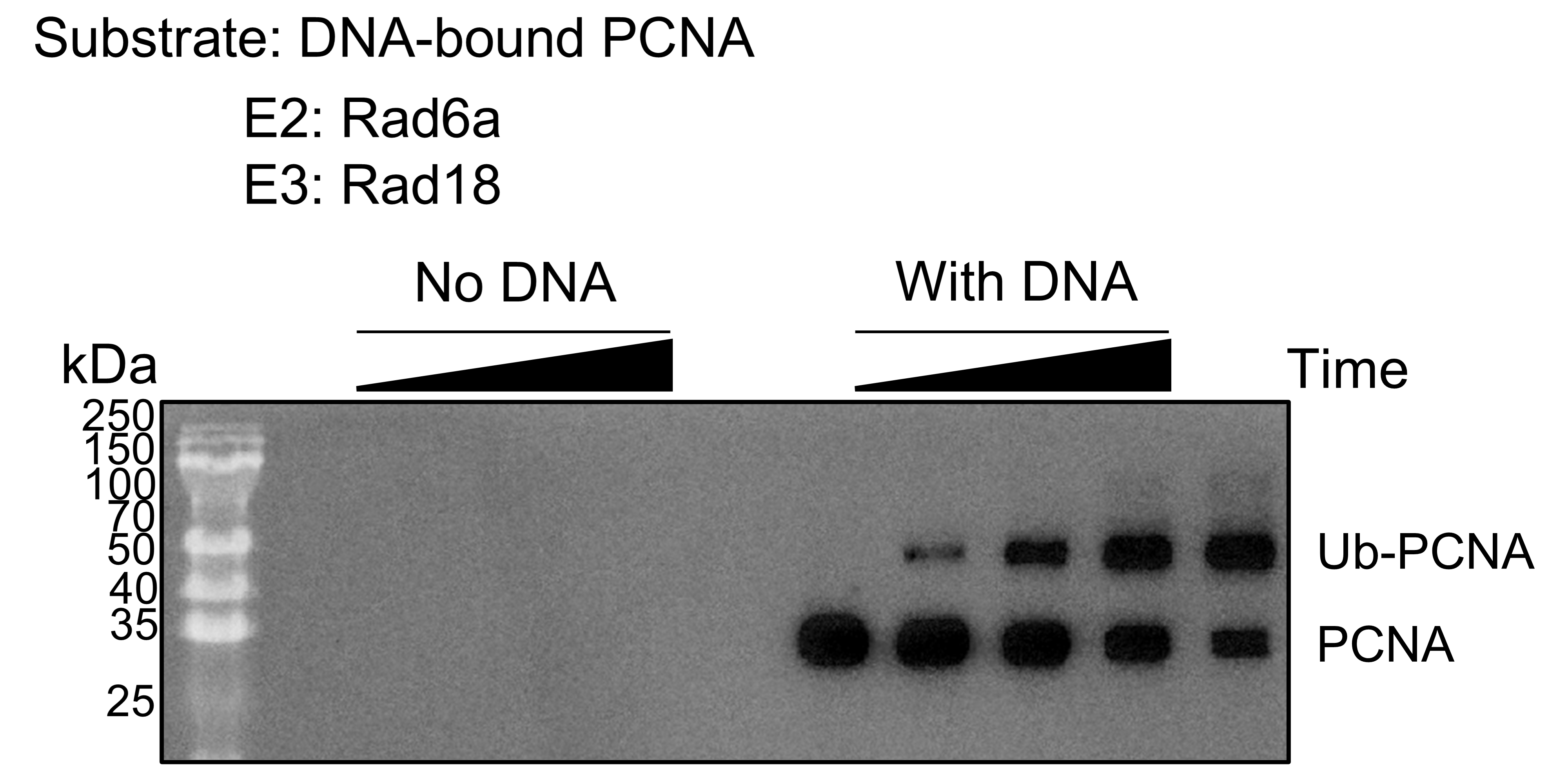

Supplement: S5 Fig — Western blot analysis for PCNA after the reaction are presented. The reactions were allowed to proceed for 0, 5, 10, 20, and 40 minutes before analysis. (TIF) [file pone.0347227.s005.tif]

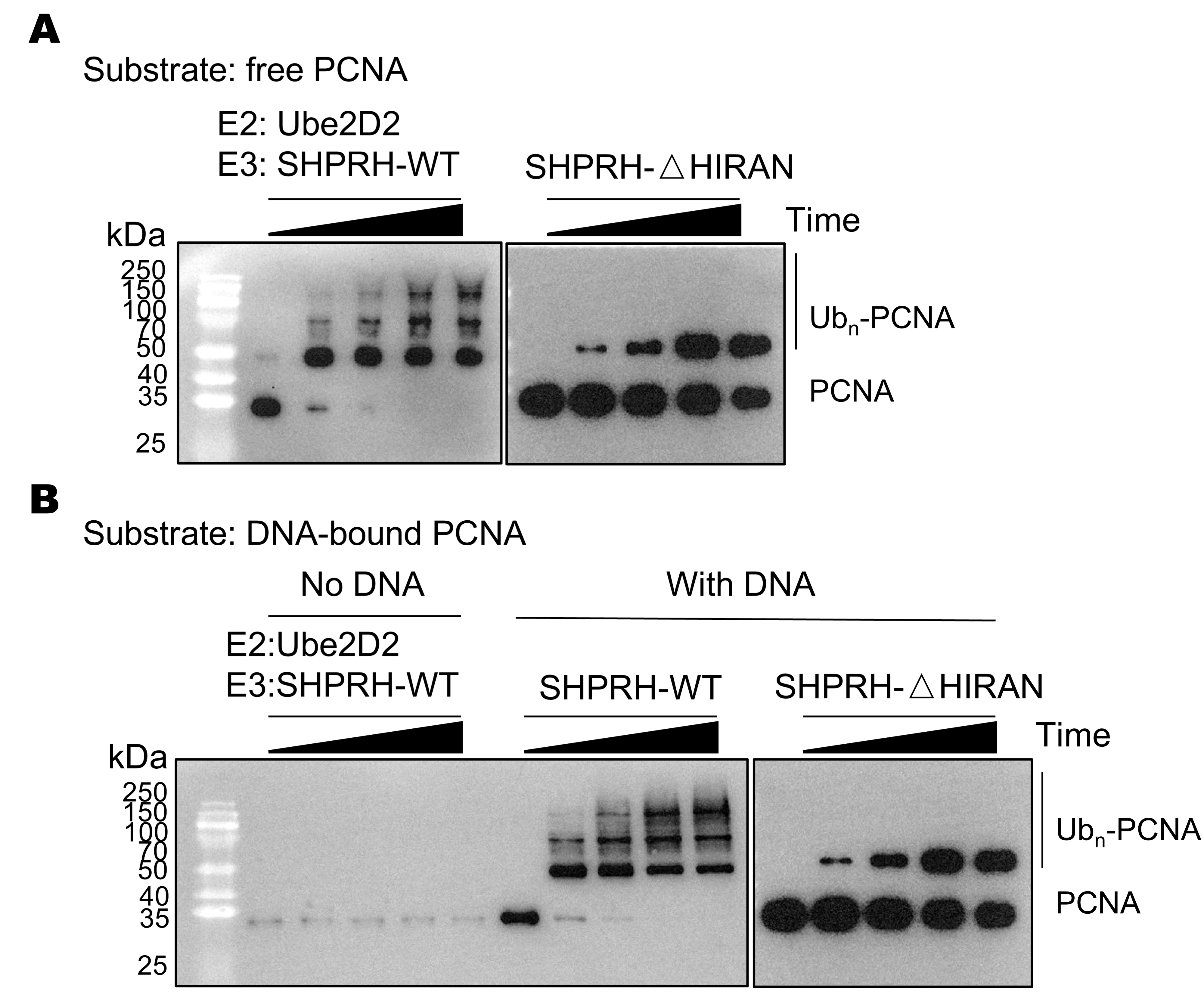

Supplement: S6 Fig — Western blot analysis of ubiquitination of free PCNA (A) and DNA-bound PCNA (B) by the wild type SHPRH or SHPRH-△HIRAN and Ube2D2 is presented. The reactions are the same as reactions presented in figure 3, except that the E2 enzyme is Ube2D2. (TIF) [file pone.0347227.s006.tif]

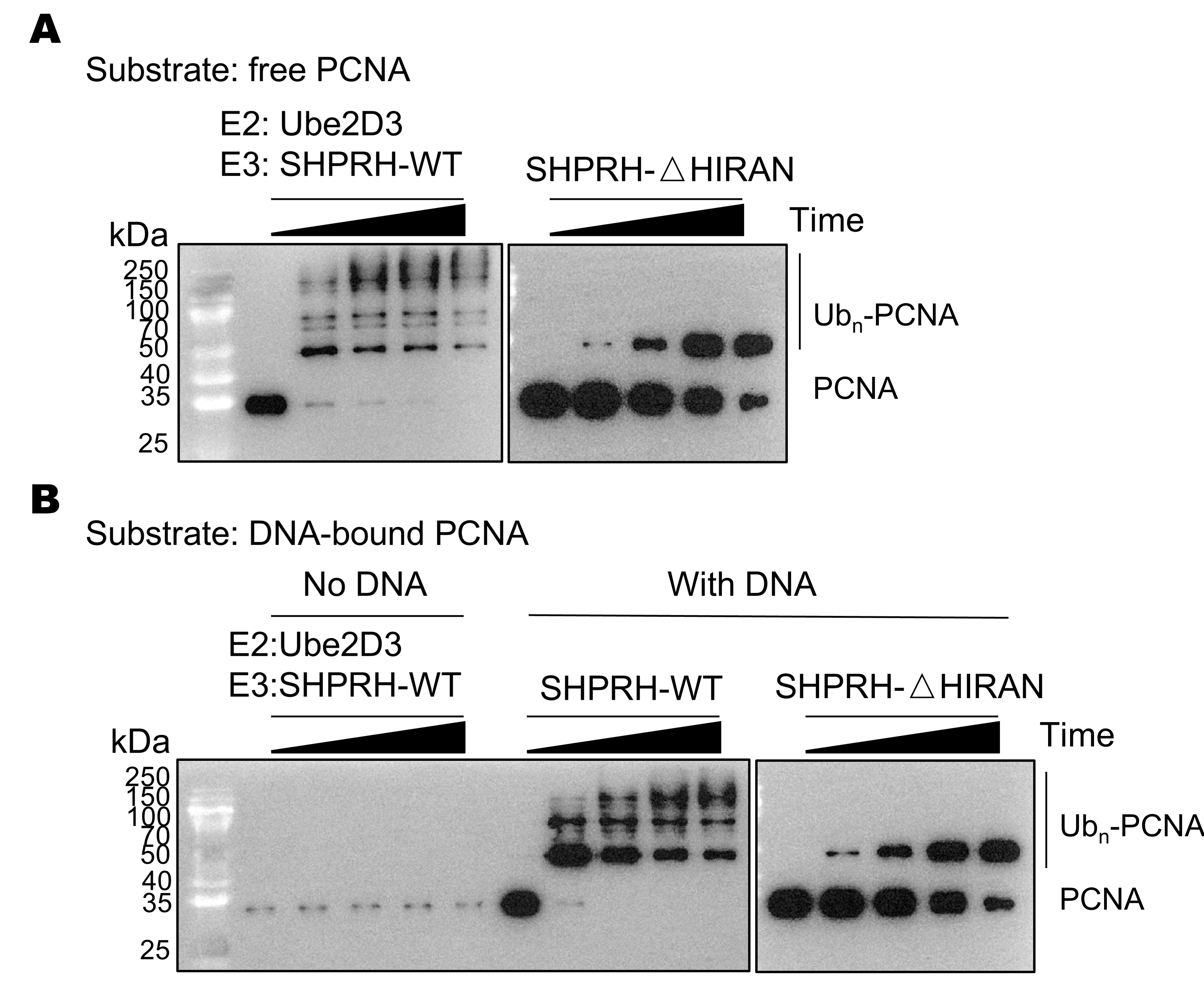

Supplement: S7 Fig — Western blot analysis of ubiquitination of free PCNA (A) and DNA-bound PCNA (B) by the wild type SHPRH or SHPRH-△HIRAN and Ube2D3 is presented. The reactions are the same as reactions presented in Fig 3, except that the E2 enzyme is Ube2D3. (TIF) [file pone.0347227.s007.tif]

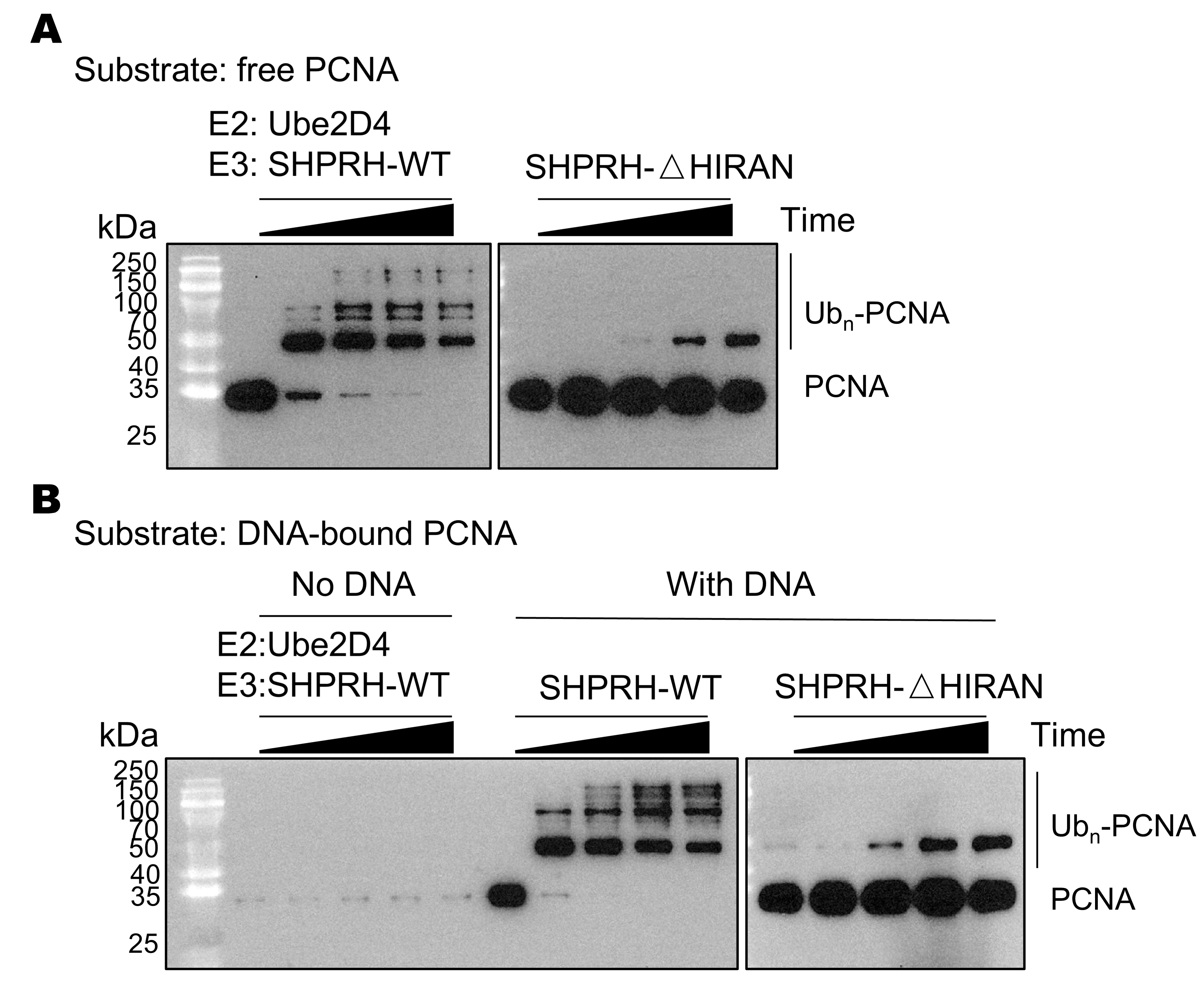

Supplement: S8 Fig — Western blot analysis of ubiquitination of free PCNA (A) and DNA-bound PCNA (B) by the wild type SHPRH or SHPRH-△HIRAN and Ube2D4 is presented. The reactions are the same as reactions presented in figure 3, except that the E2 enzyme is Ube2D4. (TIF) [file pone.0347227.s008.tif]

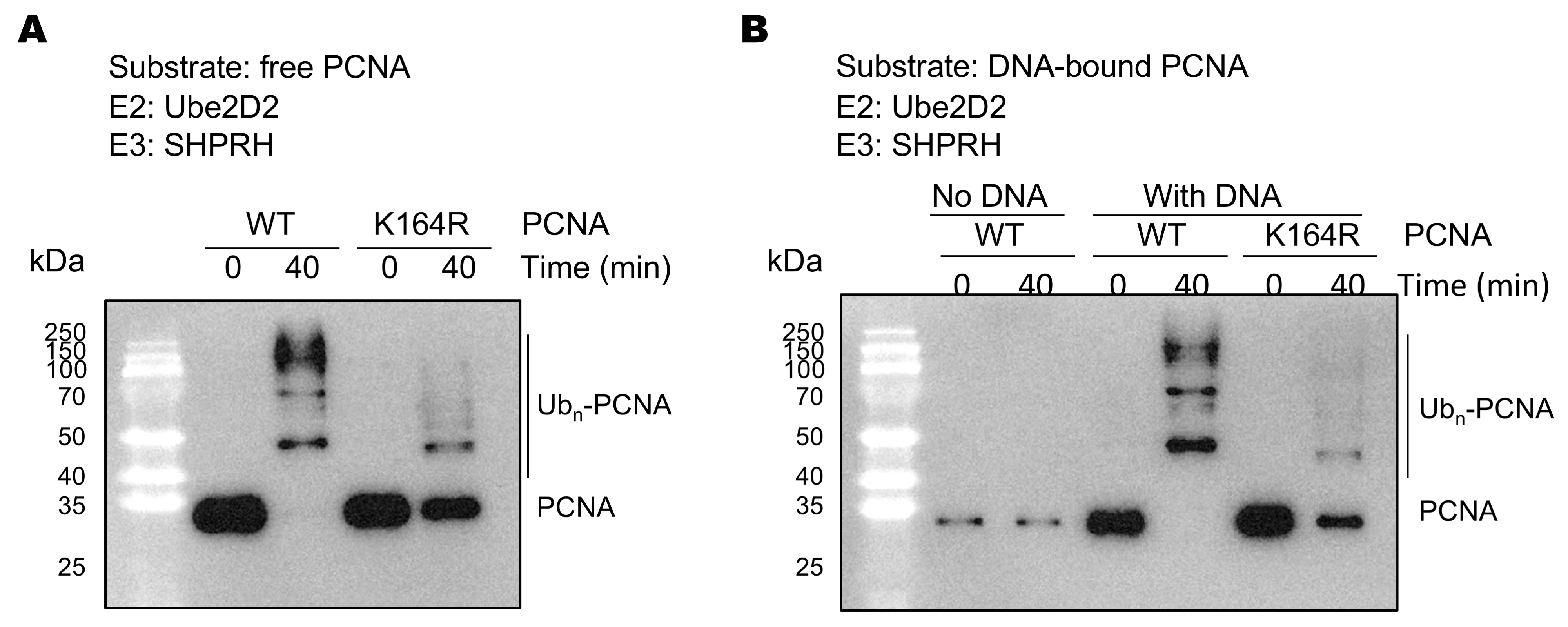

Supplement: S9 Fig — Reactions for free PCNA (A) and DNA-bound PCNA(B) are presented. The reactions are the same as those presented in figure 4, except that the E2 enzyme is Ube2D2. (TIF) [file pone.0347227.s009.tif]

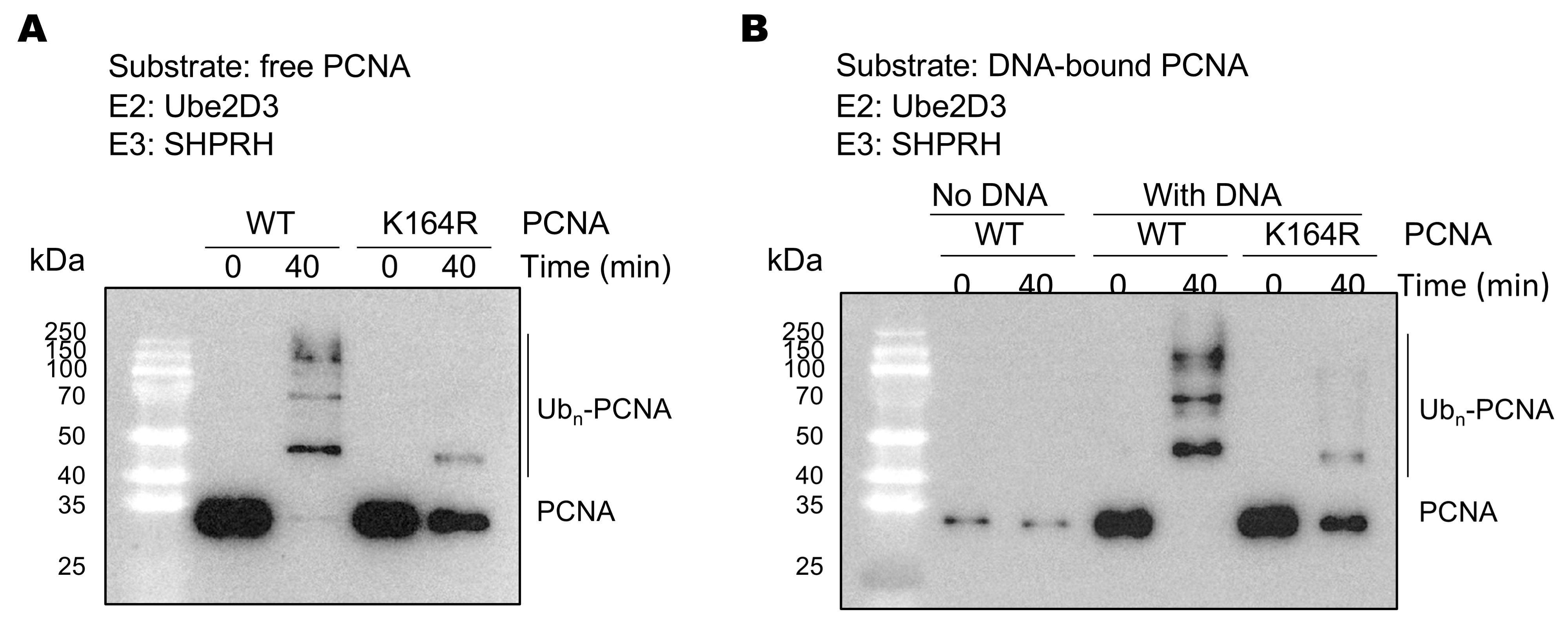

Supplement: S10 Fig — Reactions for free PCNA (A) and DNA-bound PCNA(B) are presented. The reactions are the same as those presented in figure 4, except that the E2 enzyme is Ube2D3. (TIF) [file pone.0347227.s010.tif]

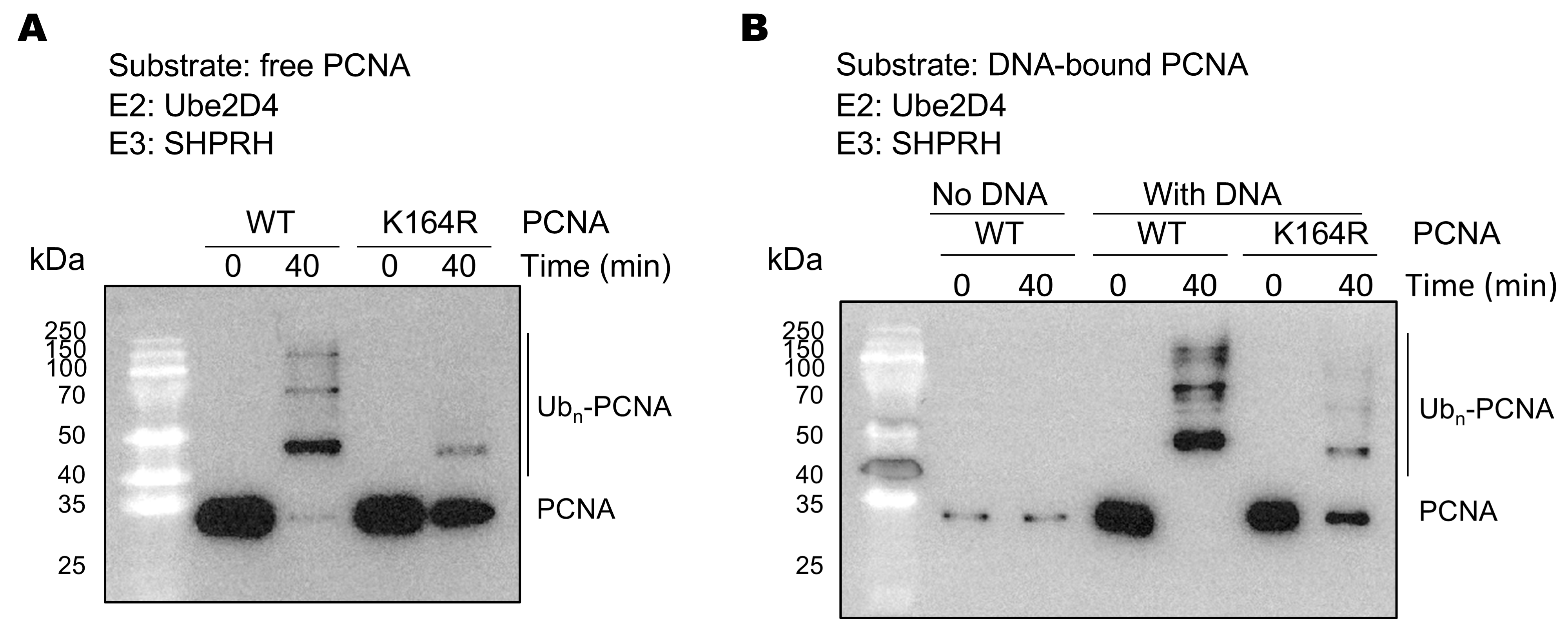

Supplement: S11 Fig — Reactions for free PCNA (A) and DNA-bound PCNA(B) are presented. The reactions are the same as those presented in figure 4, except that the E2 enzyme is Ube2D4. (TIF) [file pone.0347227.s011.tif]
