# Supplementary material for: SHPRH together with the Ube2D family of enzymes directly ubiquitinates PCNA at Lys164 in vitro
Source: PLoS One. 2026 Apr 16;21(4):e0347227. doi: 10.1371/journal.pone.0347227 (PMC13086300; doi:10.1371/journal.pone.0347227)
Supplement: S1 Table — (PDF) [file pone.0347227.s012.pdf]

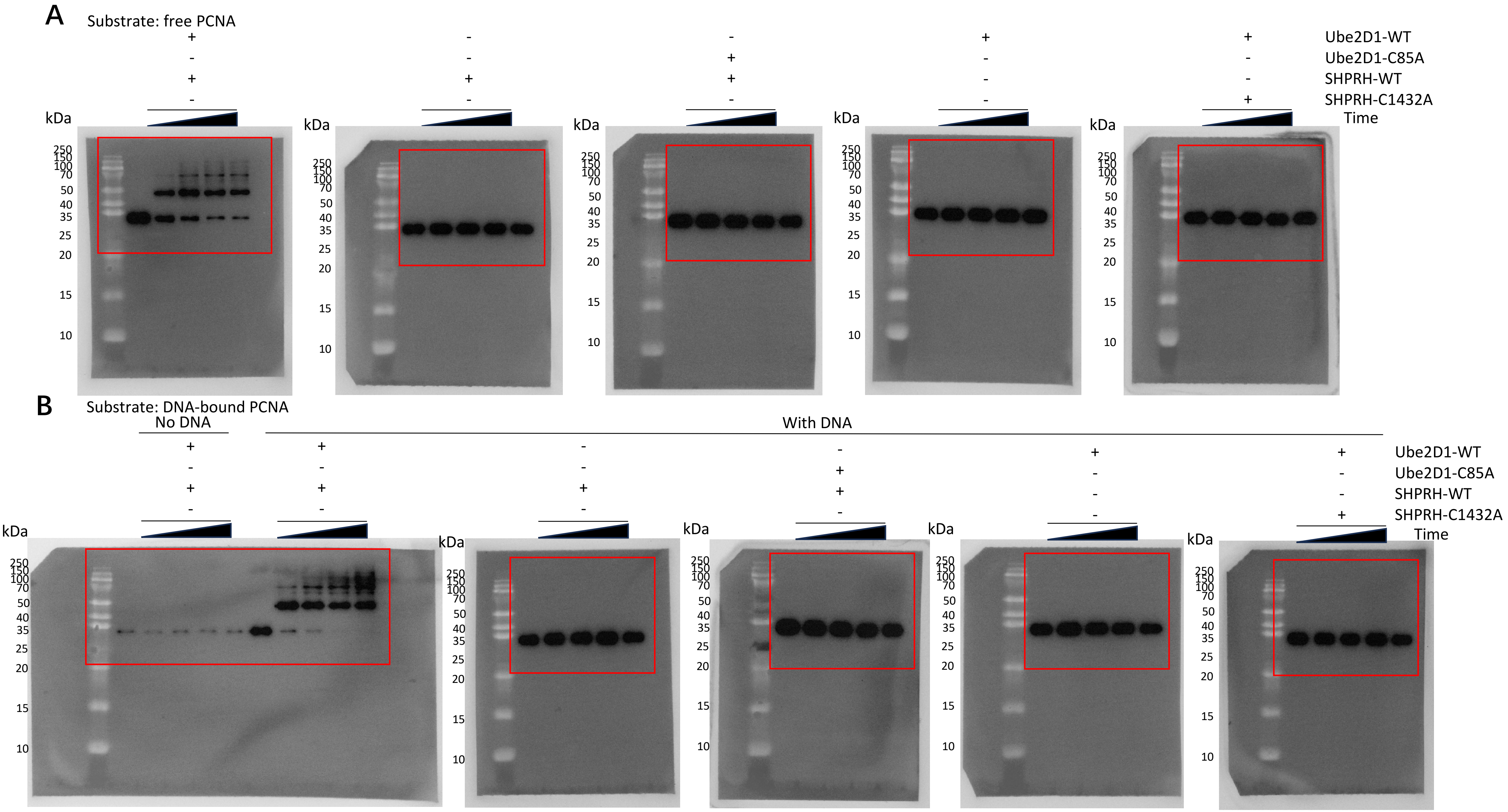

Western blot analysis of ubiquitination of free PCNA (A) and DNA-loaded PCNA (B).The reactions were allowed to proceed for 0, 5, 10, 20, and 40 minutes before termination. Marked regions are presented in Fig 1.

D

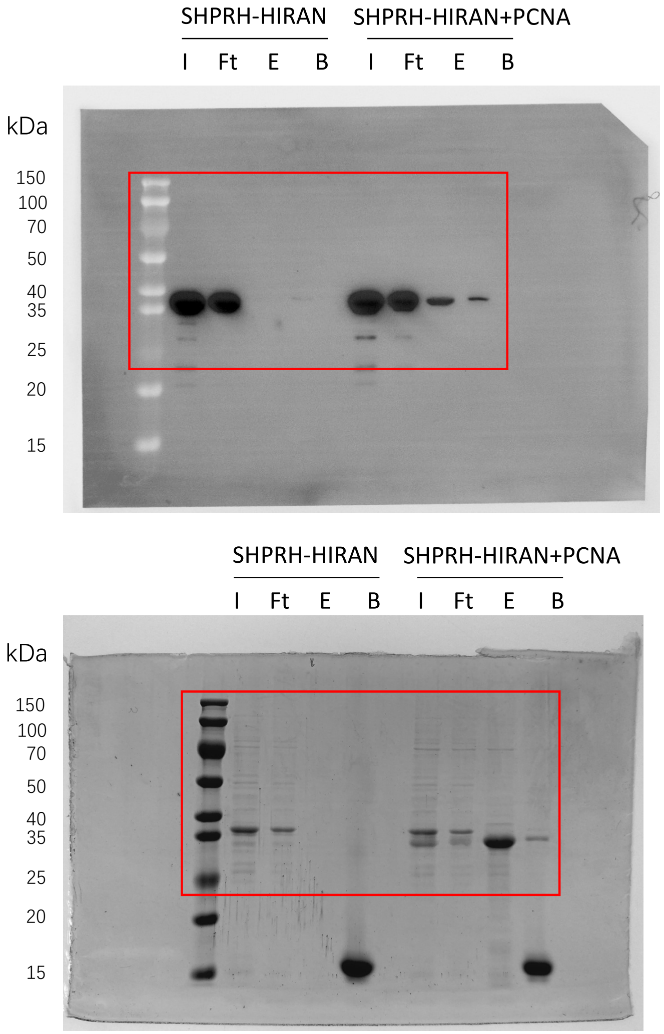

Pulldown experiments probing the interaction between PCNA and SHPRH's HIRAN domain. Western blot (for the HIRAN domain, upper panel) and SDS-PAGE (lower panel) analyses of the samples are presented. I, input protein; Ft, unbound (flowthrough) fraction; E, eluted fraction; B, the strep-tactin beads after elution.
Marked regions are presented in Fig 2D.

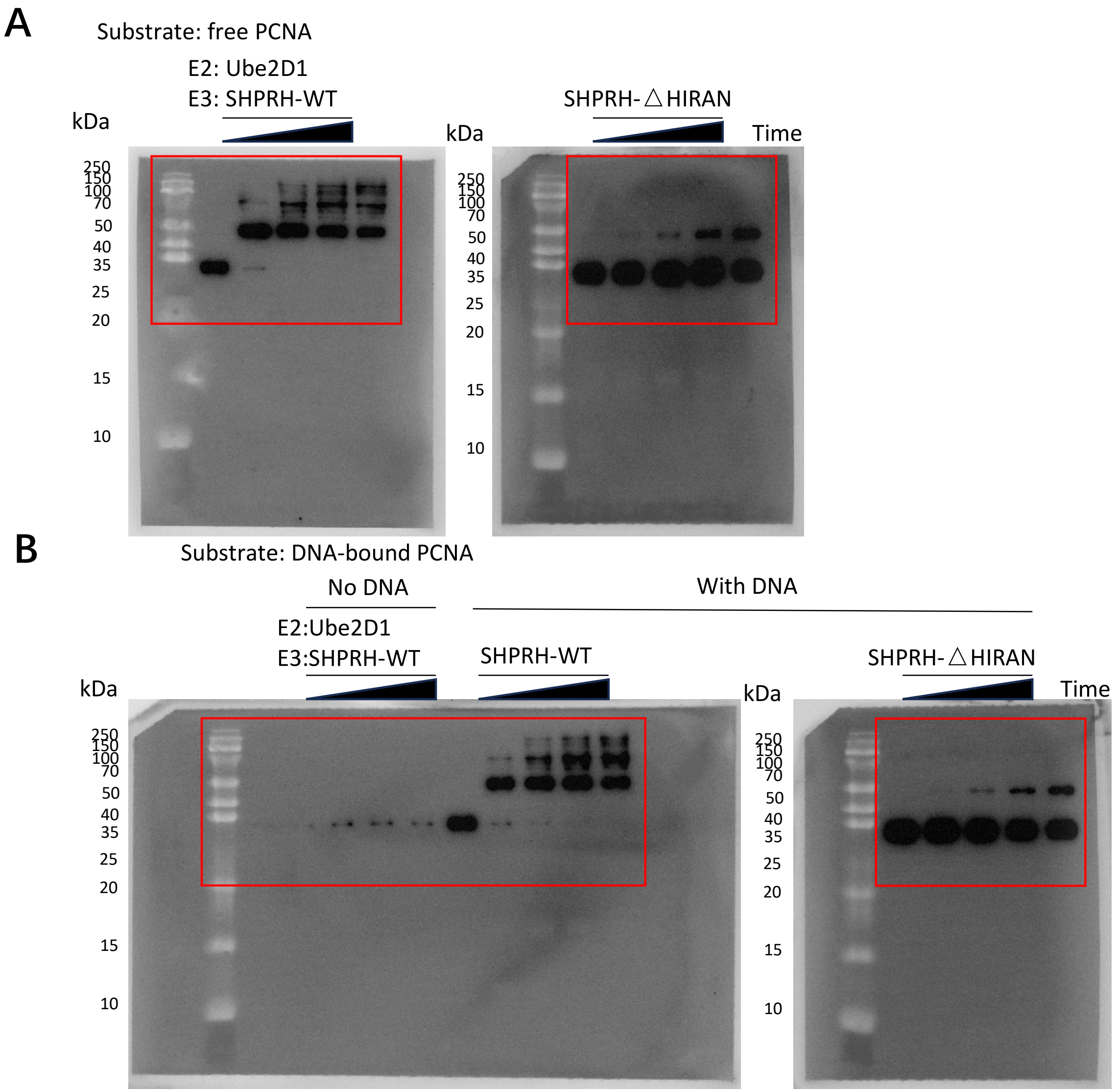

Western blot analysis of ubiquitination of free PCNA (A) and DNA-loaded PCNA (B).The reactions were allowed to proceed for 0, 5, 10, 20, and 40 minutes before termination. Marked regions are presented in Fig 3.

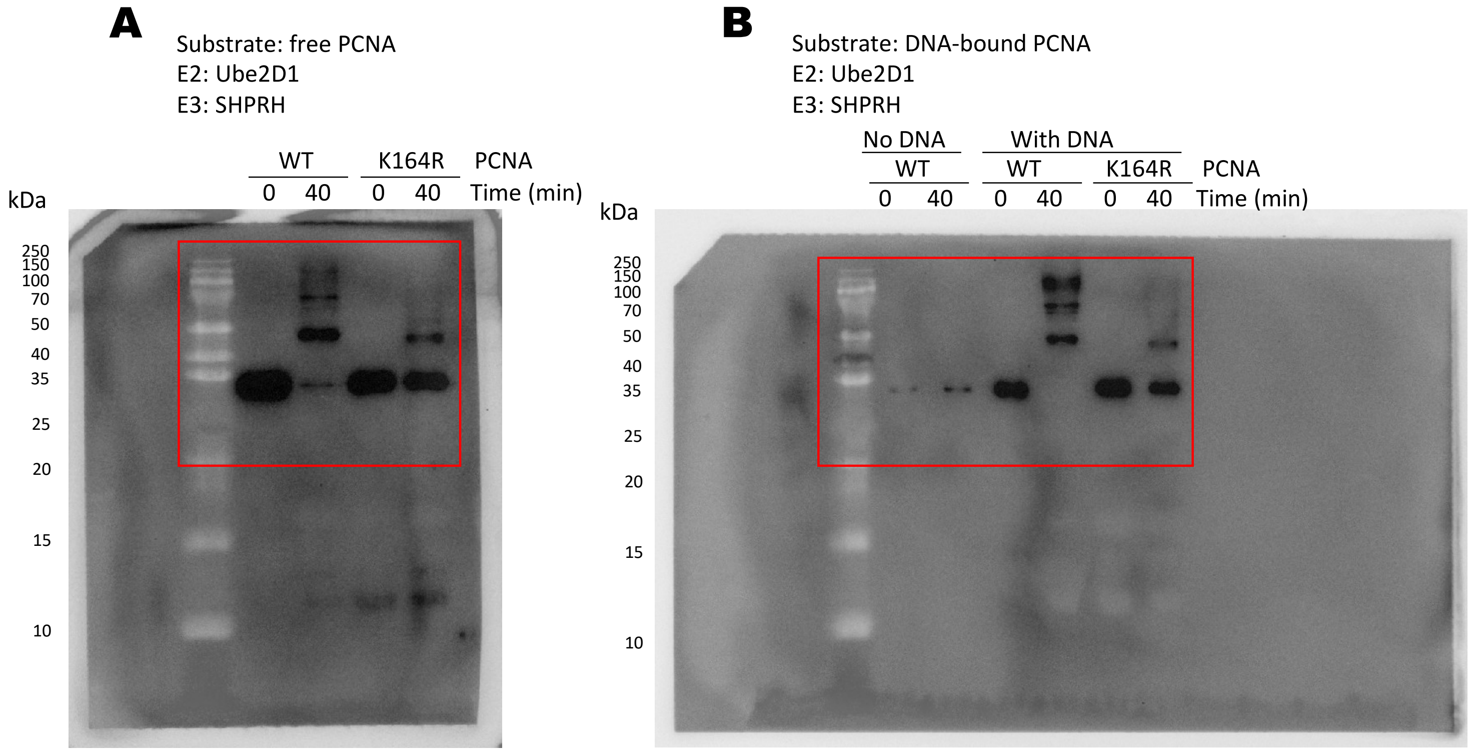

Western blot analysis of ubiquitination of free PCNA (A) and DNA-loaded PCNA (B).
Marked regions are presented in Fig 4.

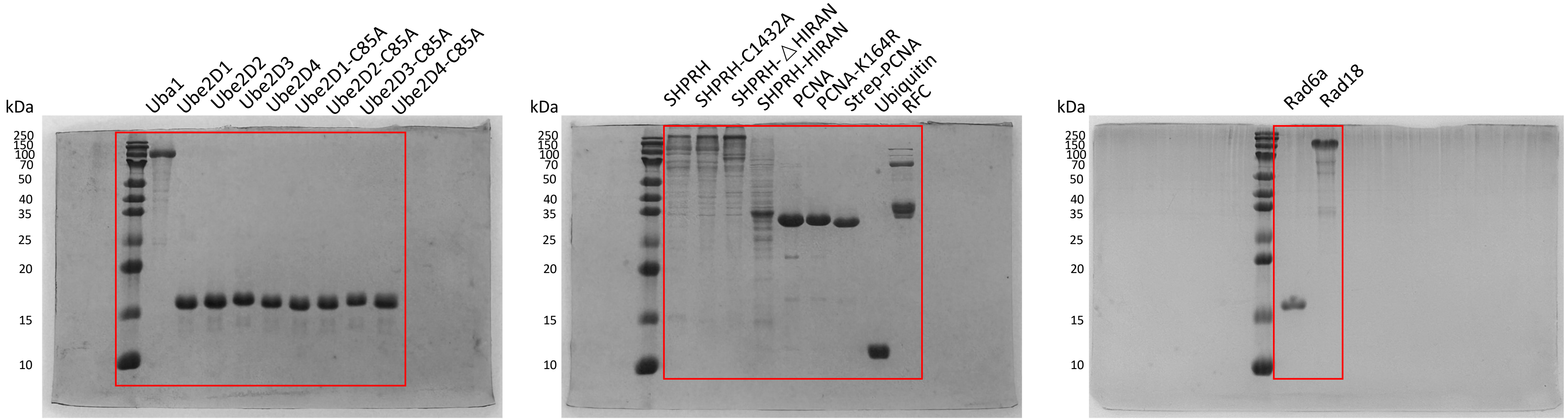

SDS PAGE analysis of the purified proteins and the RFC complex is presented.
Marked regions are presented in S1 Fig.

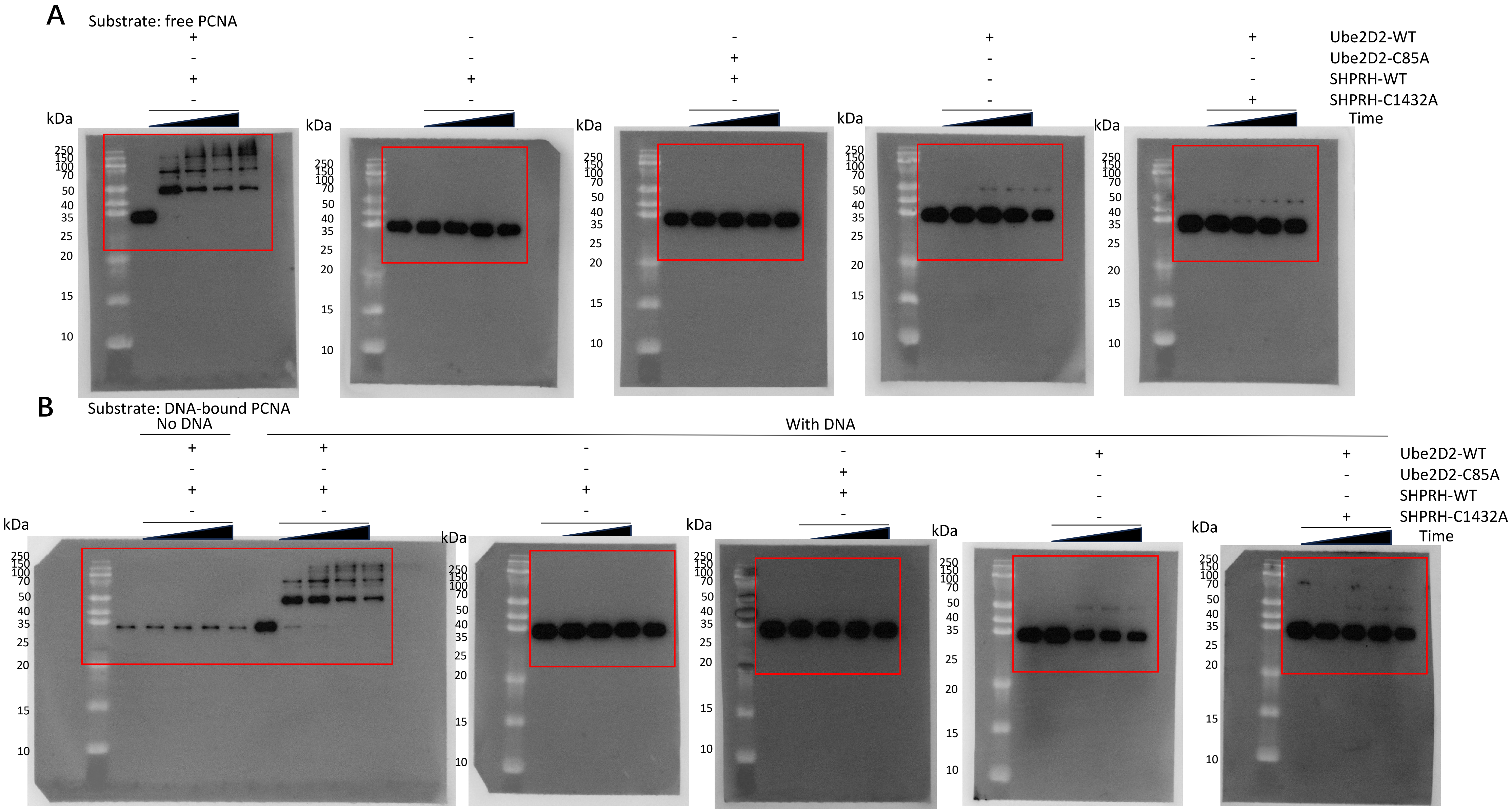

Western blot analysis of ubiquitination of free PCNA (A) and DNA-loaded PCNA (B).The reactions were allowed to proceed for 0, 5, 10, 20, and 40 minutes before termination. Marked regions are presented in S2 Fig.

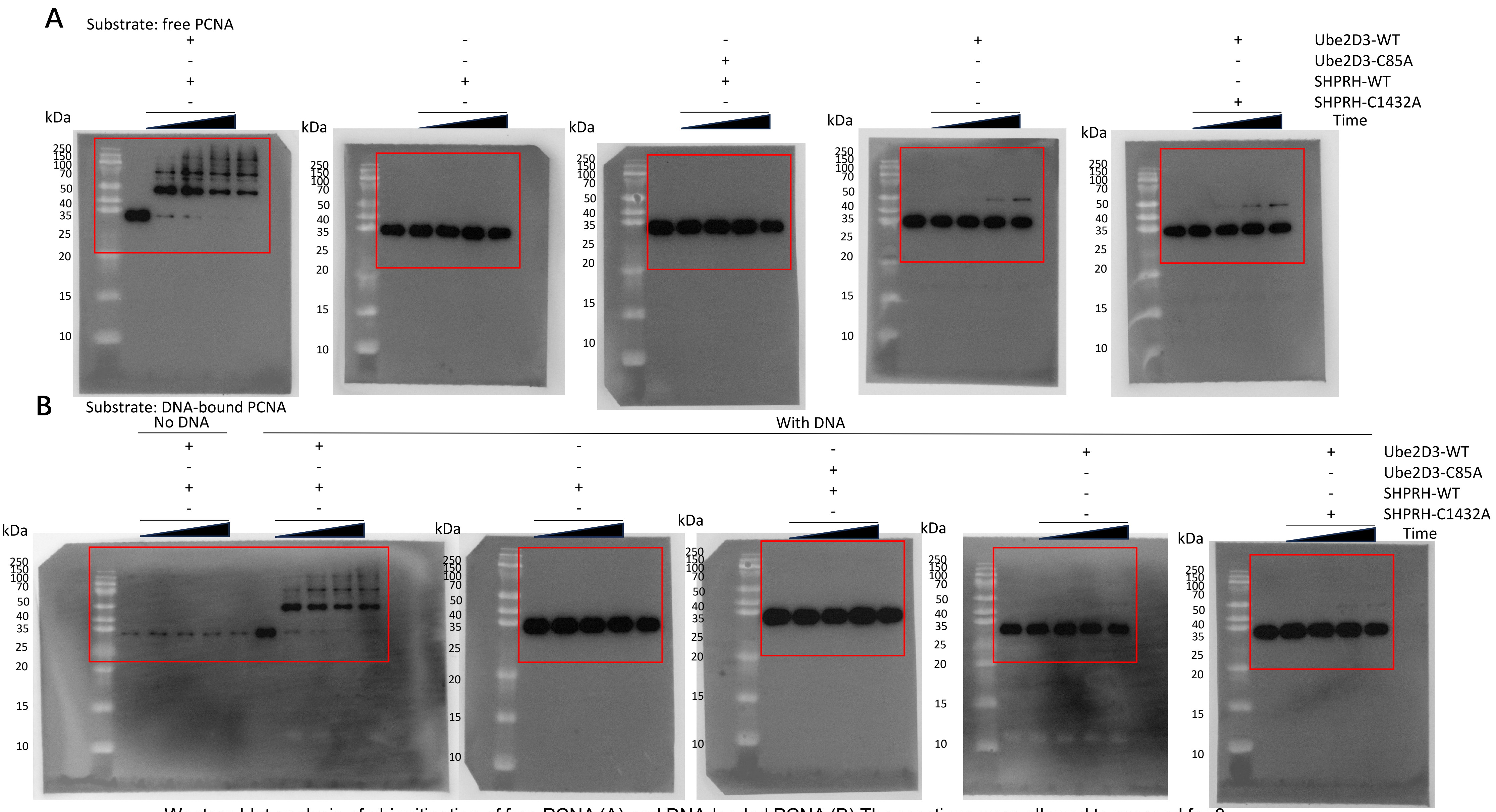

Western blot analysis of ubiquitination of free PCNA (A) and DNA-loaded PCNA (B).The reactions were allowed to proceed for 0, 5, 10, 20, and 40 minutes before termination. Marked regions are presented in S3 Fig.

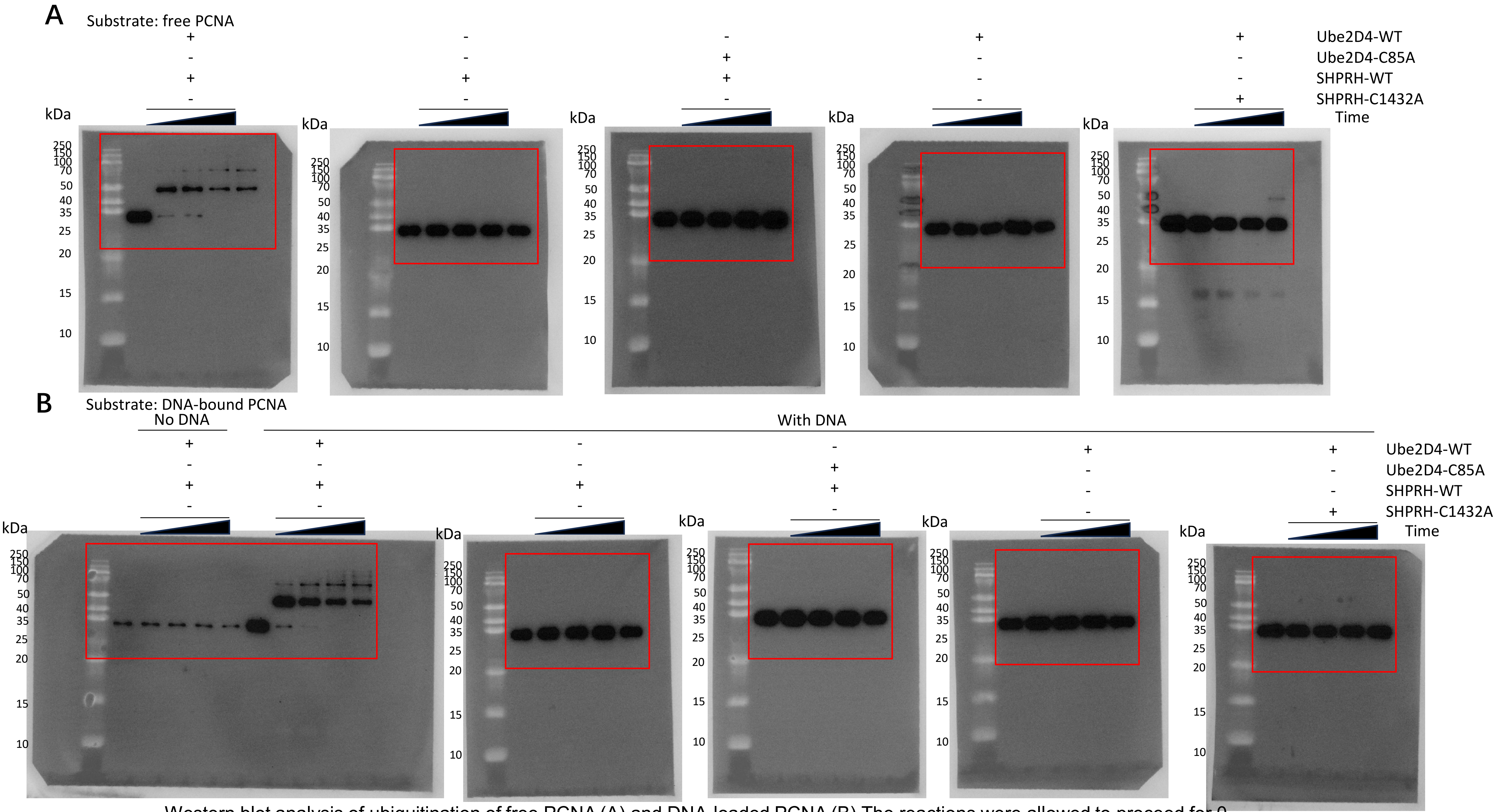

Western blot analysis of ubiquitination of free PCNA (A) and DNA-loaded PCNA (B).The reactions were allowed to proceed for 0, 5, 10, 20, and 40 minutes before termination. Marked regions are presented in S4 Fig.

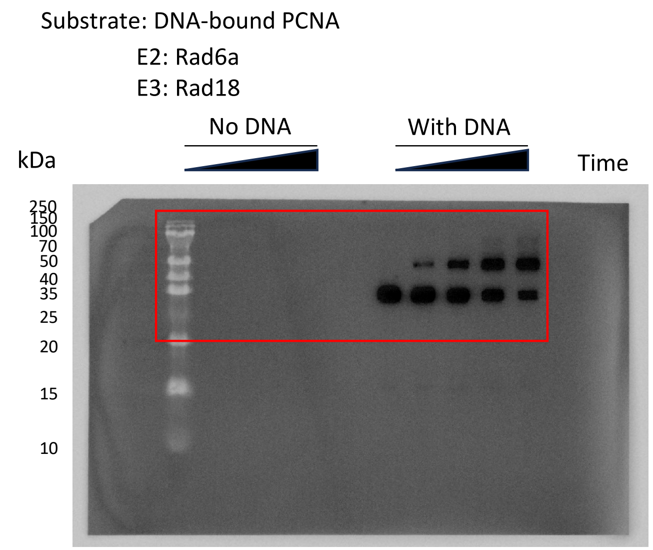

Western blot analysis for PCNA for the reaction are presented. The reactions were allowed to proceed for 0, 5, 10, 20, and 40 minutes before termination.
Marked regions are presented in S5 Fig.

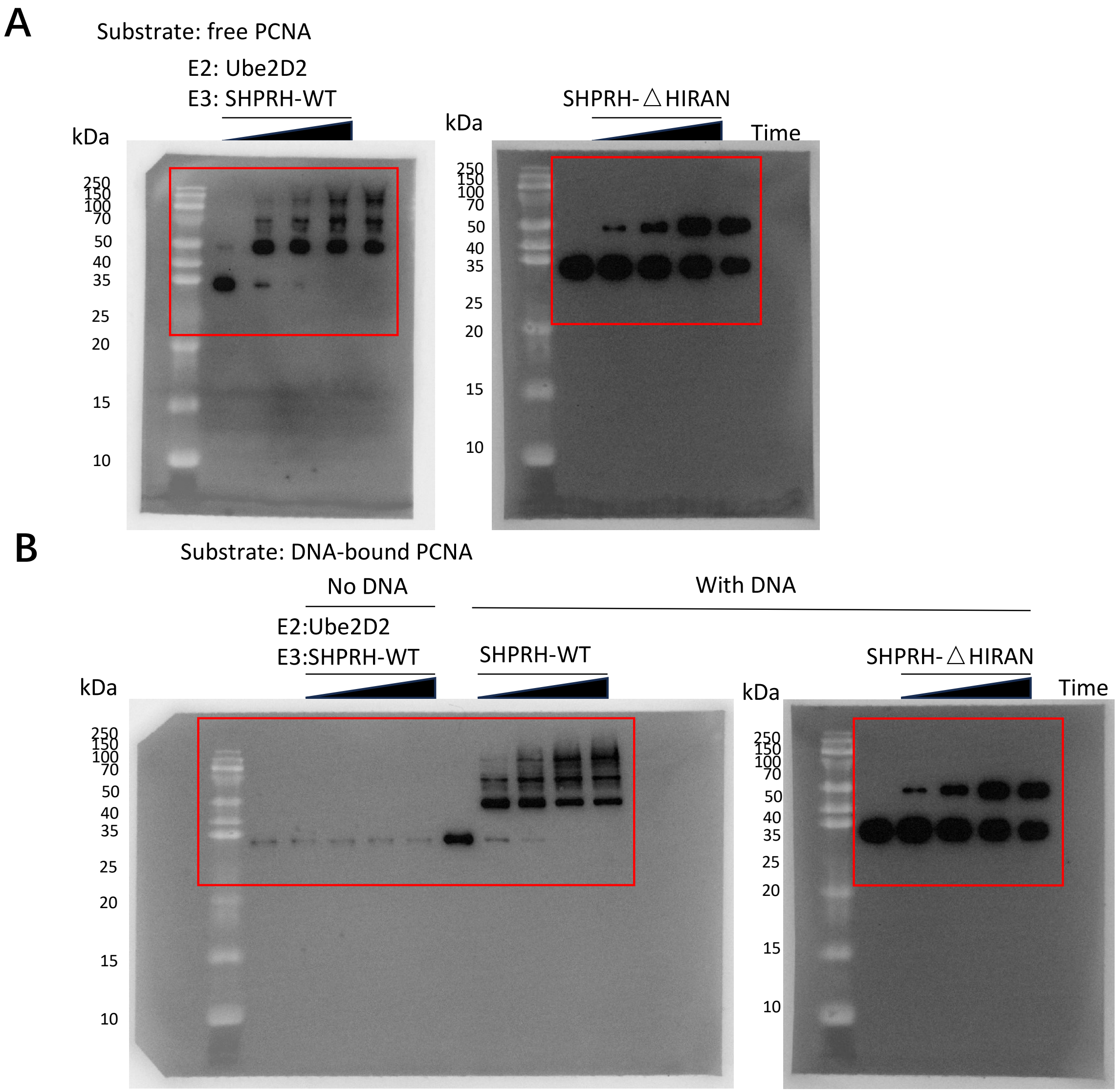

Western blot analysis of ubiquitination of free PCNA (A) and DNA-loaded PCNA (B).The reactions were allowed to proceed for 0, 5, 10, 20, and 40 minutes before termination. Marked regions are presented in S6 Fig.

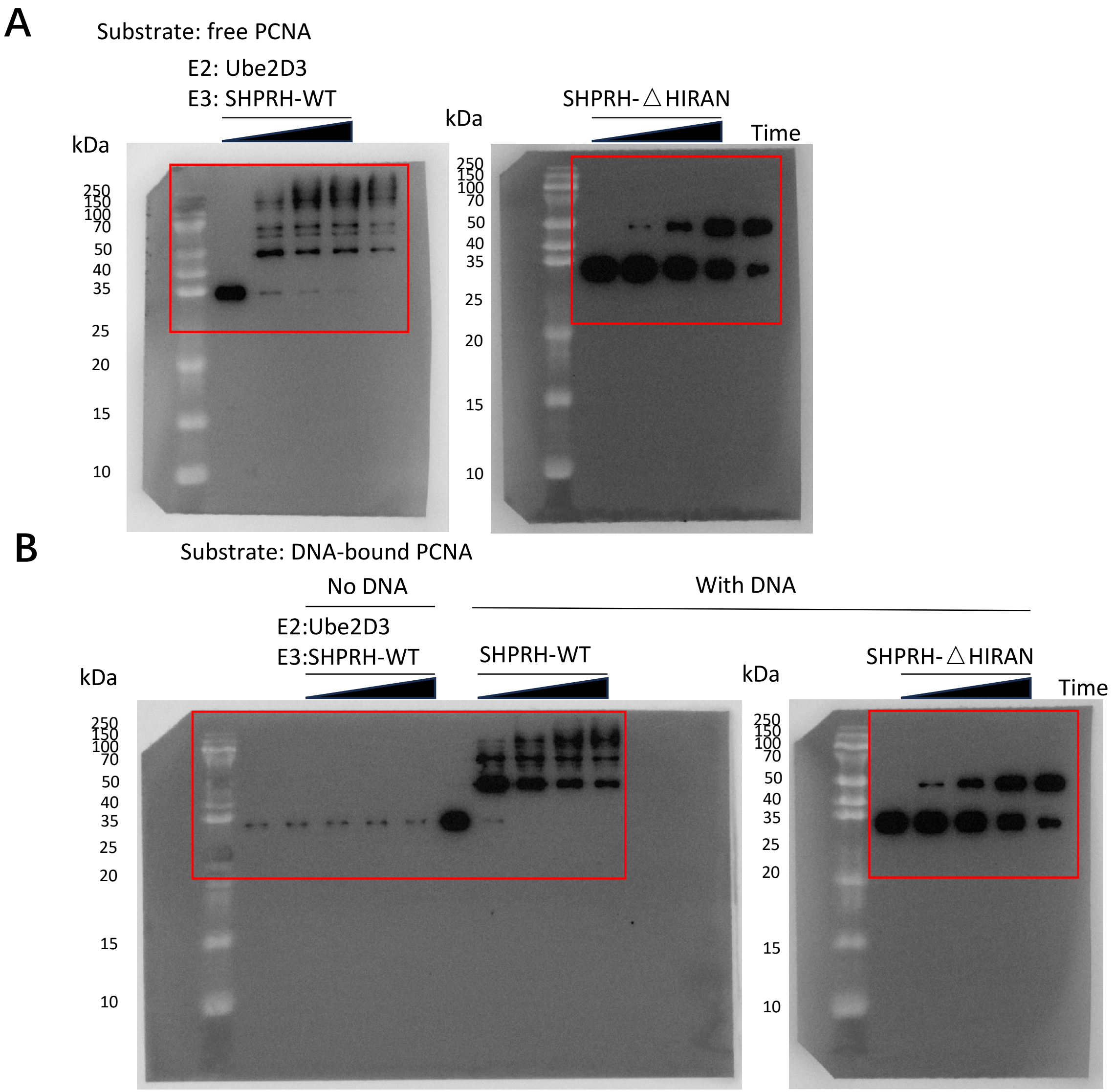

Western blot analysis of ubiquitination of free PCNA (A) and DNA-loaded PCNA (B).The reactions were allowed to proceed for 0, 5, 10, 20, and 40 minutes before termination. Marked regions are presented in S7 Fig.

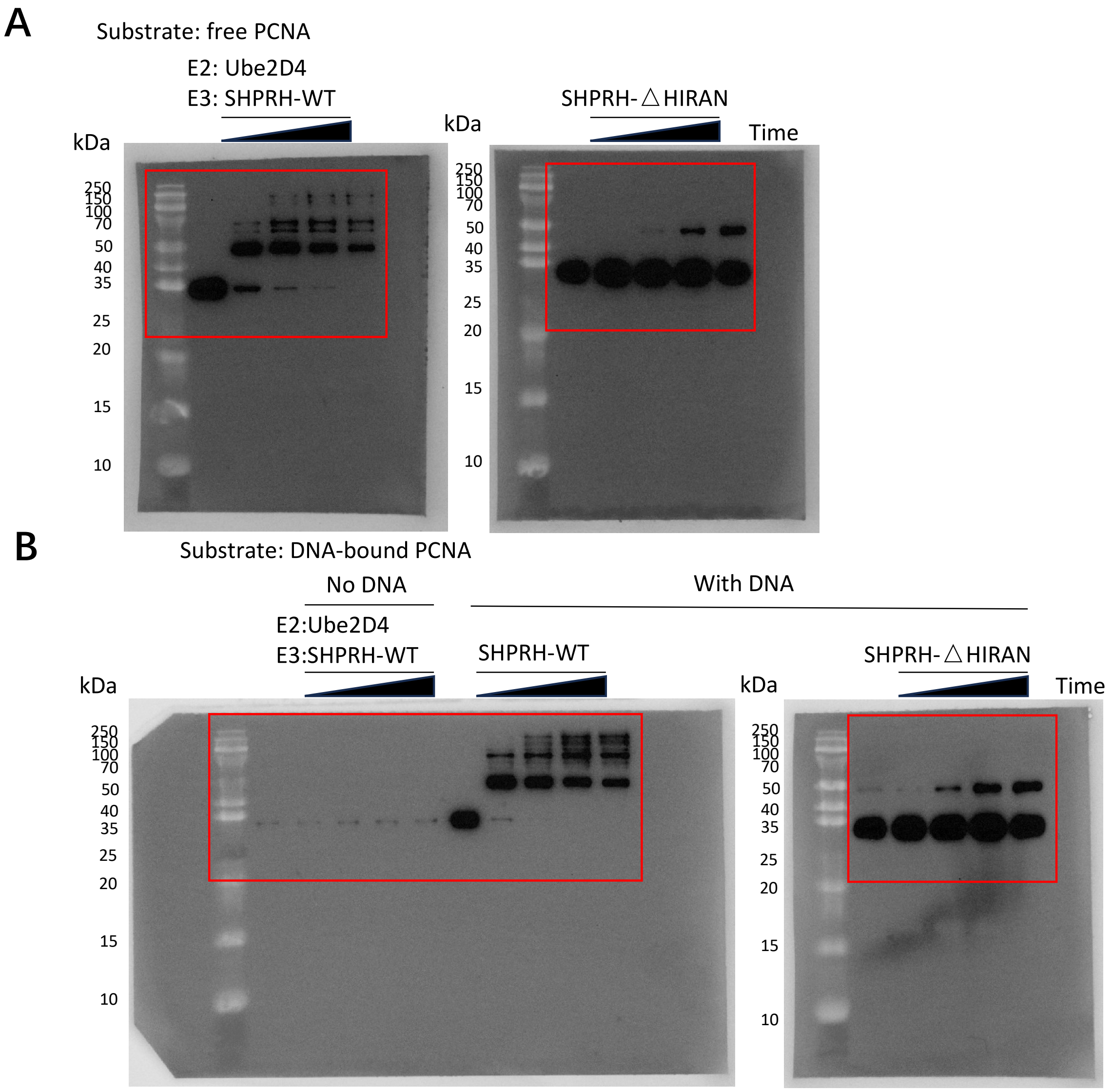

Western blot analysis of ubiquitination of free PCNA (A) and DNA-loaded PCNA (B).The reactions were allowed to proceed for 0, 5, 10, 20, and 40 minutes before termination. Marked regions are presented in S8 Fig.

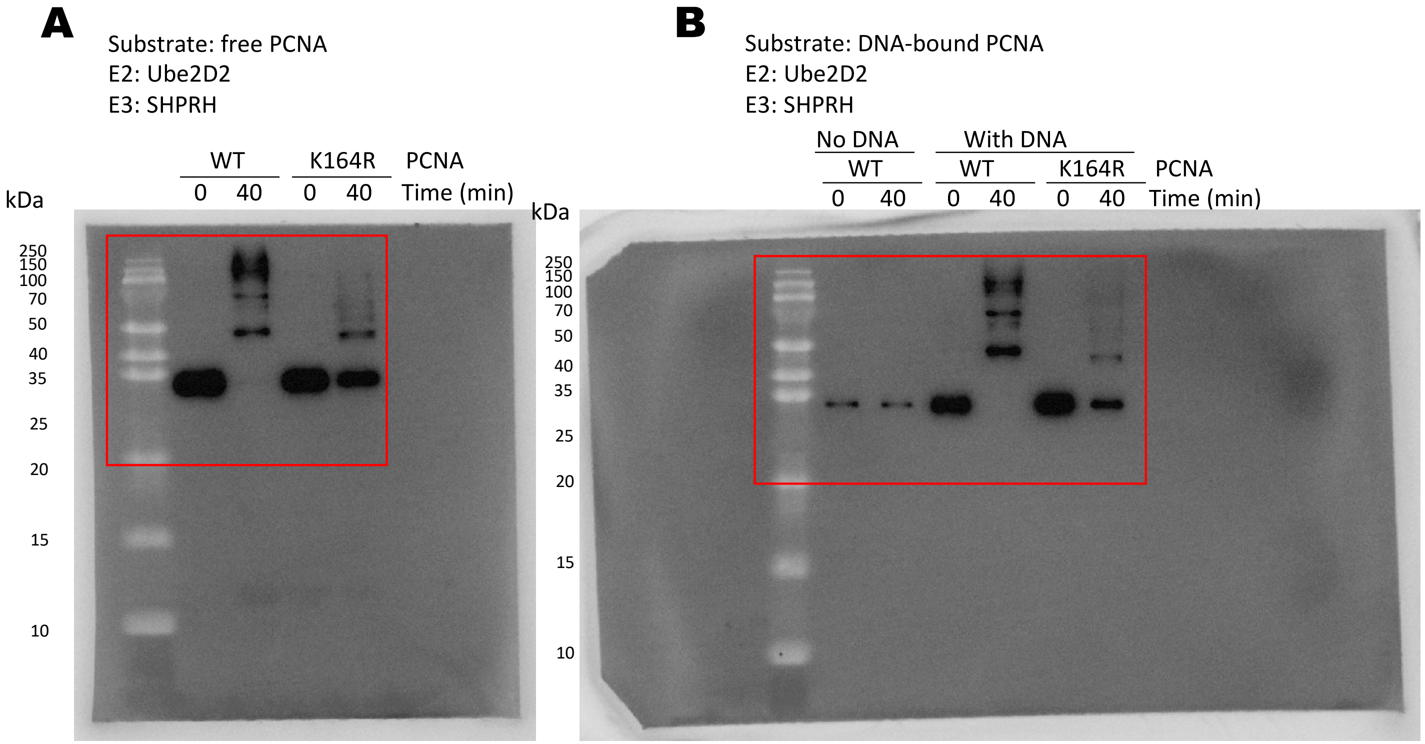

Western blot analysis of ubiquitination of free PCNA (A) and DNA-loaded PCNA (B). Marked regions are presented in S9 Fig.

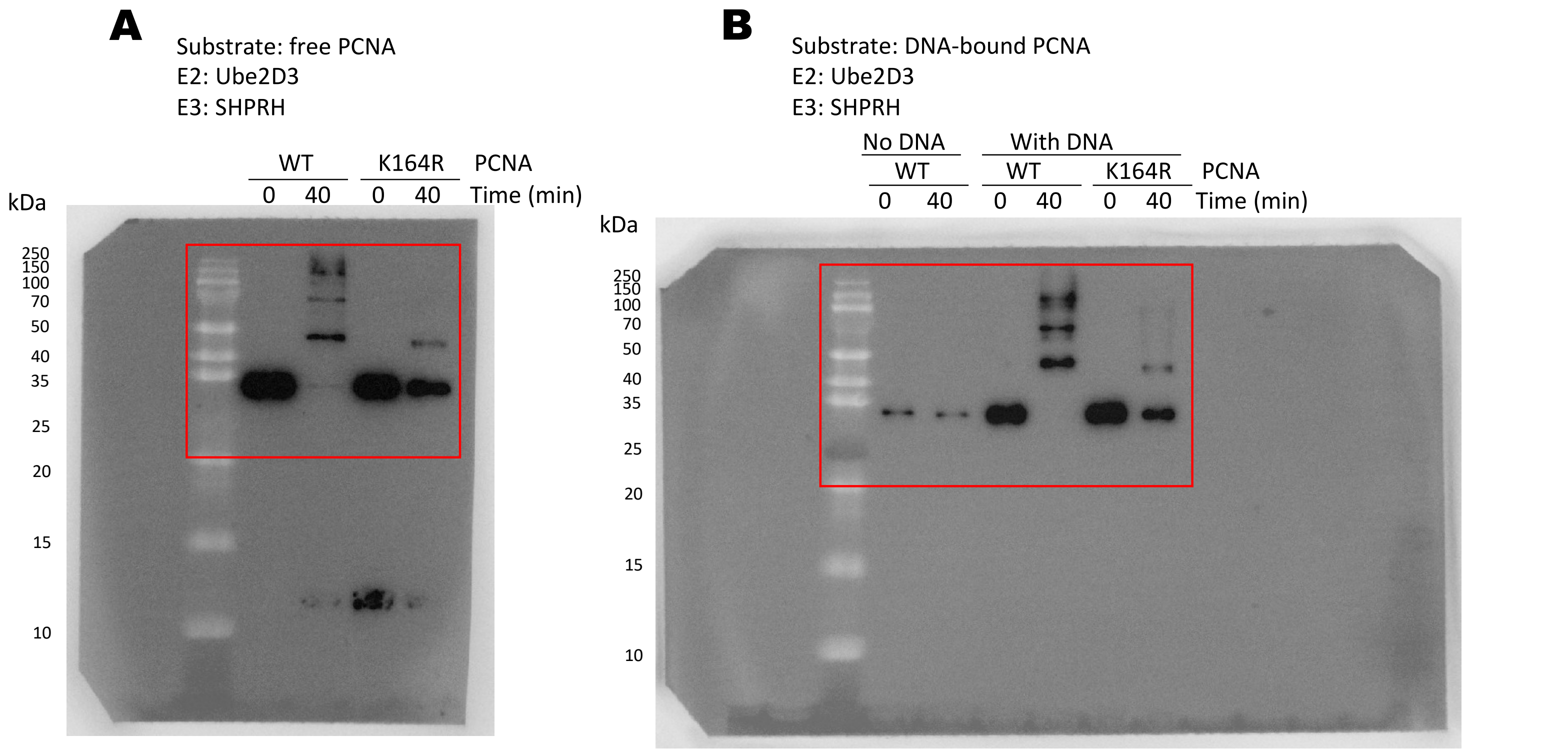

Western blot analysis of ubiquitination of free PCNA (A) and DNA-loaded PCNA (B).
Marked regions are presented in S10 Fig.

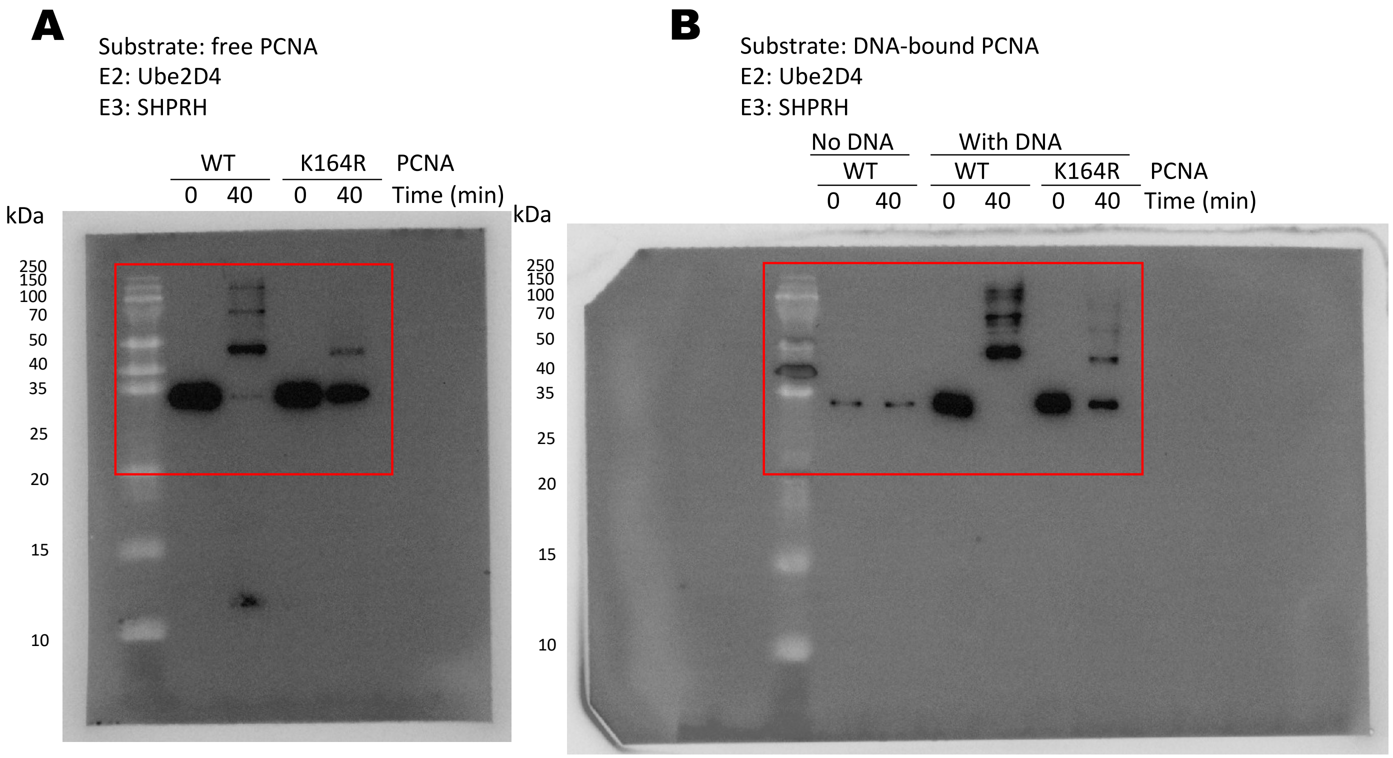

Western blot analysis of ubiquitination of free PCNA (A) and DNA-loaded PCNA (B). Marked regions are presented in S11 Fig.
